# Phosphoproteomic analysis on ovarian follicles reveals the involvement of LSD1 phosphorylation in Chicken follicle selection

**DOI:** 10.1186/s12864-023-09223-6

**Published:** 2023-03-13

**Authors:** Yanhong Zhang, Qiuyue Chen, Yuanyuan Guo, Li Kang, Yi Sun, Yunliang Jiang

**Affiliations:** 1 Shandong Provincial Key Laboratory of Animal Biotechnology and Disease Control and Prevention, College of Animal Science and Veterinary Medicine, Shandong Agricultural University, 271018 Tai’an, China; 2 College of Life Sciences, Shandong Agricultural University, 271018 Tai’an, China; 3 Experimental Center, Shandong University of Traditional Chinese Medicine, 250355 Jinan, PR China

**Keywords:** Chicken, Follicle selection, Phosphorylation, LSD1

## Abstract

**Background:**

Follicle selection in chickens refers to the process of selecting one follicle from a group of small yellow follicles (SY, 6–8 mm in diameter) for development into 12–15 mm hierarchal follicles (usually F6 follicles), which is controlled by sex hormones including follicle-stimulating factor (FSH), estrogen and progesterone. Follicle selection is a critical process impacting egg production in chicken, therefore, is the focus of many studies. Phosphorylation is important for the proper function of proteins, thus, needs to be analyzed by proteomic level.

**Result:**

In this study, we compared the phosphoproteomes of SY and F6 follicles in laying hens and identified 2,386 phosphoproteins and 5,940 phosphosites, of which 4,235 sites of 1,963 phosphoproteins were quantified. From SY to F6 follicles, 190 phosphorylation sites of 149 proteins changed significantly, among which the phosphorylation level of lysine demethylase 1 A (LSD1) at the conserved 54^th^ serine (LSD1Ser54p) was significantly upregulated in F6 follicles compared to SY follicles (*p* < 0.05); however, the expression of chicken LSD1 were not significantly different on both mRNA and protein levels. LSD1Ser54p is mainly located in the nucleus of both SY and F6 follicles, and was higher in F6 follicles than that of SY follicles revealed by both immunofluorescence and Western blotting. LSD1Ser54p level increased after treatment with 5 ng/mL and 10 ng/mL of FSH in the theca cells and the granulosa cells of pre-hierarchal follicles, and with 50 ng/mL in granulosa cells of hierarchal follicles. In the theca cells of hierarchal follicles, estrogen stimulated the level of LSD1Ser54p in a dosage-dependent manner, and in granulosa cells of pre-hierarchal follicles, 10 ng/mL of estrogen increased LSD1Ser54p expression. Treatment with 50 ng/mL of progesterone increased LSD1Ser54p expression in theca cells of pre-hierarchal follicles, and with 10 to 100 ng/ml enhanced LSD1Ser54p expression in the granulosa cells of hierarchal follicles.

**Conclusion:**

The expression dynamics of LSD1Ser54p in follicles from SY to F6 and its regulation by sex hormones suggest that it is involved in chicken follicle selection.

**Supplementary Information:**

The online version contains supplementary material available at 10.1186/s12864-023-09223-6.

## Background

Egg production is an important trait for both laying lines and broiler breeders. Hens are also medical model for human reproduction disorder. At hatch, approximately 480,000 oocytes are estimated to exist in the chick ovary, and during the adult life of the bird, only a few hundred oocytes are selected to reach maturity and ovulate [1]. In the domestic chicken, the functionally mature ovary contains hundreds of white cortical follicles that are 1–5 mm in diameter, 5–6 small yellow follicles (SY) of 6–8 mm in diameter and 5–6 large yellow preovulatory follicles that are 9–40 mm in diameter [1]. Follicle selection in chicken refers to the selection of one follicle from a pool of growing follicles, usually the SY follicles with a diameter of 6–8 mm, into a preovulatory hierarchy of follicles ranging in a diameter of 9–40 mm. The follicle being selected begins to become dominant and continues maturation until it ovulates [2].

Follicle selection is a critical process of egg laying, which occurs when the hen reaches sexual maturation and begins to lay the first egg, and when a single follicle is selected on a daily or near-daily basis (also referred to as cyclic recruitment) for the duration of the laying sequence [3]. In a reproductively efficient hen, such as Hy-Line brown commercial egg laying hens, the largest preovulatory follicle will ovulate each day and one 6–8 mm SY follicle will be selected to replace it and replenish the preovulatory follicle pool [2]. Two of the most important characteristics of follicle selection in chicken are granulosa cell differentiation and the initiation of progesterone synthesis, accompanied by the increased expression of steroidogenic acute regulatory protein (*StAR*) and cytochrome P450scc (*CYP11A1*) genes, which is regulated by paracrine, endocrine and autocrine factors [4–6].

A working model of chicken follicle selection [3] states that, before follicle selection, the differentiation of follicular granulosa cell is suppressed by active.

mitogen-activated protein kinase (MAPK) signaling *via* extracellular signal-regulated kinases (ERK1, ERK2); while at follicle selection, the inhibitory effects of MAPK/ERK signaling and FSHR/VPAC (vasoactive intestinal peptide receptor) desensitization mediated *via* β –arrestin are released. During follicle selection, the granulosa cell layer in one follicle from the 6–8 mm cohort initiates and sustains several critical processes including transcription of *StAR* and *CYP11A* within the steroidogenic pathway by protein kinase A (PKA) /cAMP signaling [3]. This model is further surported by the study showing that the 6–8 mm SY follicle exhibits the greatest response to follicle-stimulating hormone (FSH) and bone morphogenetic protein 6 (BMP6) [7] and that the expression of follicle-stimulating hormone receptor (FSHR) varies among the multiple SY follicles isolated from a single chicken ovary, one of which exhibits a higher expression level of FSHR compared to the others [8] and is likely to be selected into follicular hierarchy. Comparison of transcriptomes between chicken SY follicles differing in the mRNA expression of FSHR identified hundreds of differentially expressed genes, of which the Wnt4 signaling pathway is significantly enriched and involved in chicken follicle selection by stimulating granulosa cell proliferation and steroidogenesis [9]. Further whole transcriptome sequencing analysis on such SY follicles differing in FSHR expression identified differentially expressed miRNAs and lncRNAs genes, and reveals an inhibitory role of sosondowah ankyrin repeat domain family member A during follicle selection [10].

We previously compared the transcriptomes, proteomes and transcriptomes of 6–8 mm SY follicles and the smallest hierarchal follicles (F6) in laying hens and found several differentially expressed genes/proteins including VLDLR that are related to chicken follicle selection [11]. Protein phosphorylation is the most common post-translational modification of proteins, which regulates many biological processes such as signal transduction, gene expression and cell cycle through protein phosphorylation and dephosphorylation [12, 13]. In recent years, the development of high-throughput quantitative phosphorylated proteome technology provides an opportunity to elaborate the function of phosphorylated proteins on a large scale. In particular, the labeling technology with tandem mass labeling not only allows the comparison of a variety of different samples in mass spectrometry based experiments, but also can evaluate a large number of cellular proteins. Therefore, in a single experiment, it can simultaneously identify and quantify phosphorylation sites [14]. In this study, we further analyzed the phosphoproteomics of SY follicles and F6 follicles of laying hens during the peak period of egg laying in Hy-Line brown chicken. We found that the phosphorylation of LSD1 at the 54th serine (LSD1Ser54p) was significantly upregulated in F6 follicles and was regulated by sexual hormones in the theca and granulosa cells of pre-hierarchal and hierarchal follicles, suggesting an involvement of LSD1Ser54p in chicken follicle selection.

## Results

### Phosphoprotein Identification by TMT-labeling and LC-MS/MS analysis

**Fig. 1:**
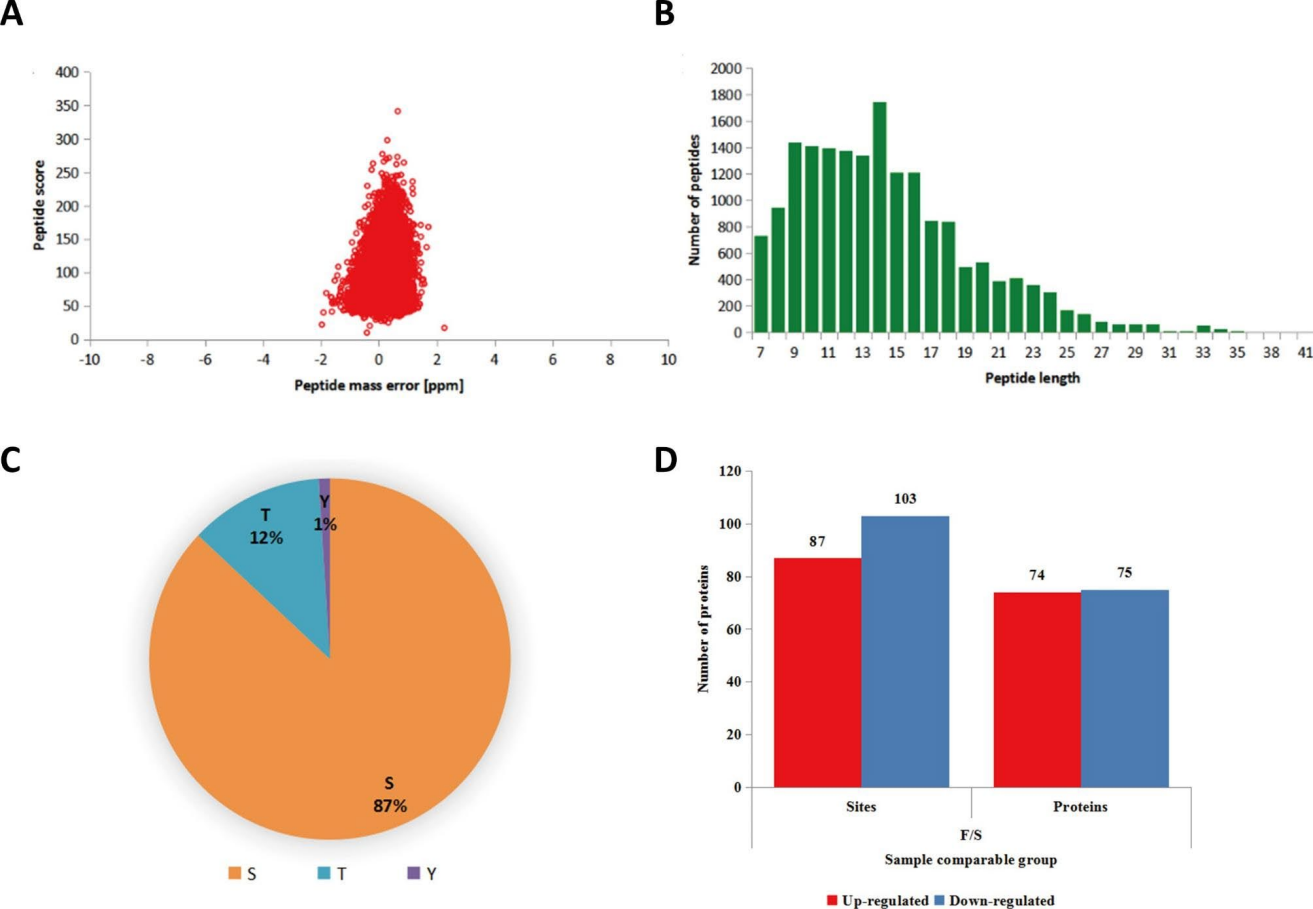
Data quality of phosphoproteomic analysis. **(A)** Mass error distribution of all identified peptides. **(B)** The length distribution of the majority of the peptides. **(C)** Statistics of differential phosphorylation site corresponding proteins. S: serine T: Threonine Y: tyrosine. **(D)** The histogram of differentially expressed phosphoproteins (DEPs) between chicken SY follicles and F6 follicles

The proteins extracted from SY follicles and F6 follicles were used for TMT labeling and HPLC fractionation followed by LC-MS/MS analysis. The first step was to validate the MS data. The distribution of the peptide mass error was close to zero, and most of the absolute values were less than 10 ppm, indicating that the mass accuracy of the MS data was compliant with the requirements for phosphoprotein identification **(**Fig. 1A**)**. The length of most peptides was between eight and 16 amino acids, which was in agreement with the general characteristics of tryptic peptides **(**Fig. 1B**)**. According to the criteria of localization probability > 0.75, totally 2,180 phosphoproteins containing 4,415 phosphosites were identified. Among these phosphoproteins, 3,576 sites of 1,851 phosphoproteins were quantified, of which 87% were phosphorylated at serine, 12% at threonine, and 1% at tyrosine residues **(**Fig. 1C**)**. According to the relative levels, the quantified proteins were divided into two categories: proteins with a quantitative ratio over 1.5 were considered upregulated, and proteins with a quantitative ratio less than 1/1.5 were considered downregulated. In the F6 follicles, the phosphorylation levels at 190 phosphosites of 149 phosphoproteins changed significantly as compared with those in the SY follicles (p < 0.05), among which 87 were upregulated and 103 were downregulated, respectively **(**Fig. 1D**)**.

### Gene Ontology and KEGG pathway analyses

**Fig. 2:**
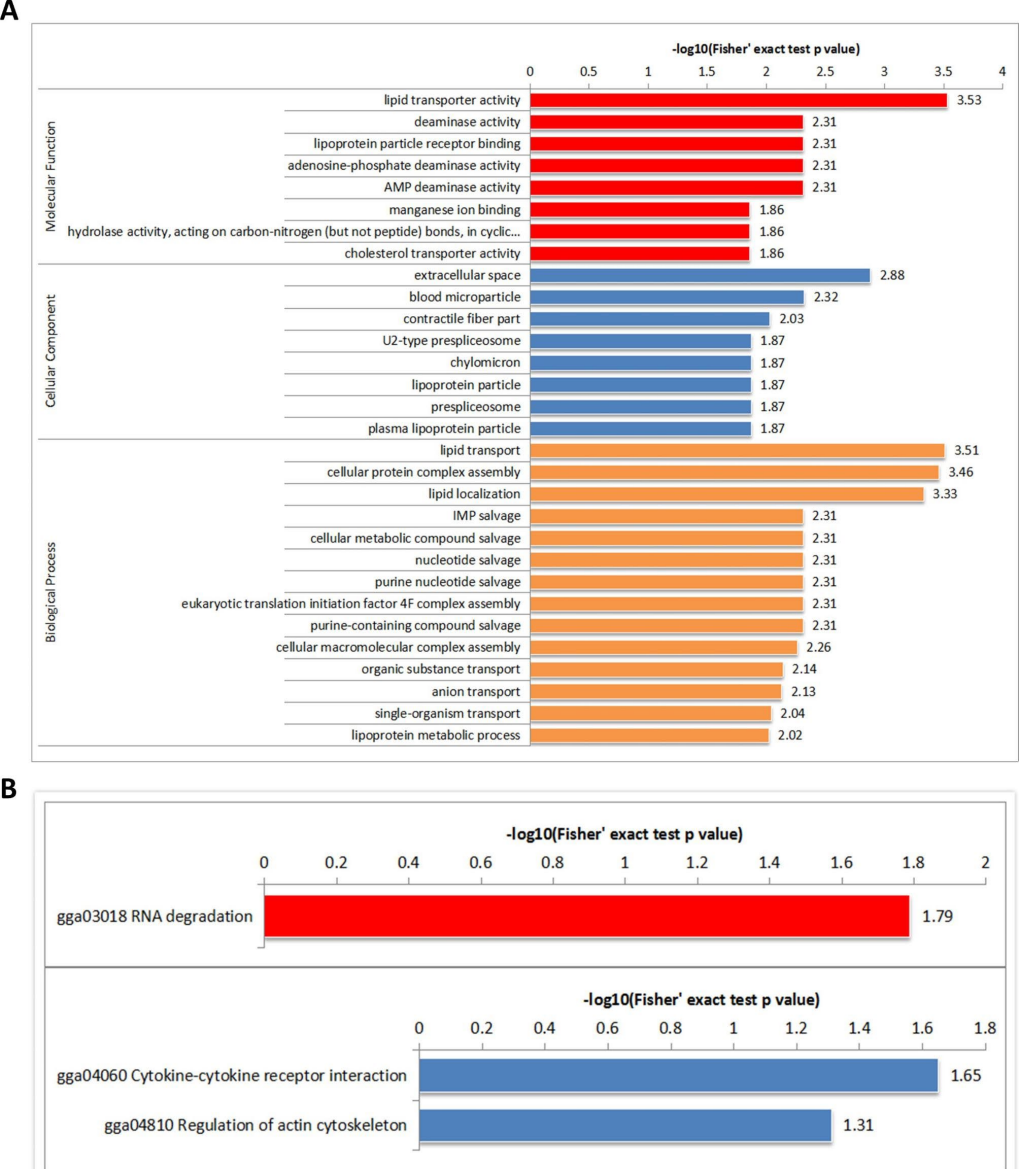
GO and KEGG enrichment analysis of differentially expressed proteins (DEPs) between small yellow follicles and F6 follicles. **(A)** GO enrichment analysis of proteins with correspondingly differential phosphorylation sites. **(B)** KEGG enrichment analysis of proteins with correspondingly differential phosphorylation sites

Proteins were classified by GO annotation into three categories: biological process (BP), cellular compartment (CC) and molecular function (MF). For each category, a two-tailed Fisher’s exact test was employed to test the enrichment of the differentially expressed proteins (DEPs) against all identified proteins. The GO with a corrected *p*-value < 0.05 is considered significant. GO enrichment analysis revealed that the top two MF categories are lipid transporter activity and deaminase activity, the top two CC were extracellular space and blood microparticle, and the top two BP categories were lipid transport and cellular protein complex assembly **(**Fig. 2A**)**. Three KEGG pathways were significantly enriched [15–17], including RNA degradation pathway, cytokine-cytokine receptor interaction pathway and regulation of actin cytoskeleton pathway **(**Fig. 2B**)**.

### Motif analysis

**Fig. 3:**
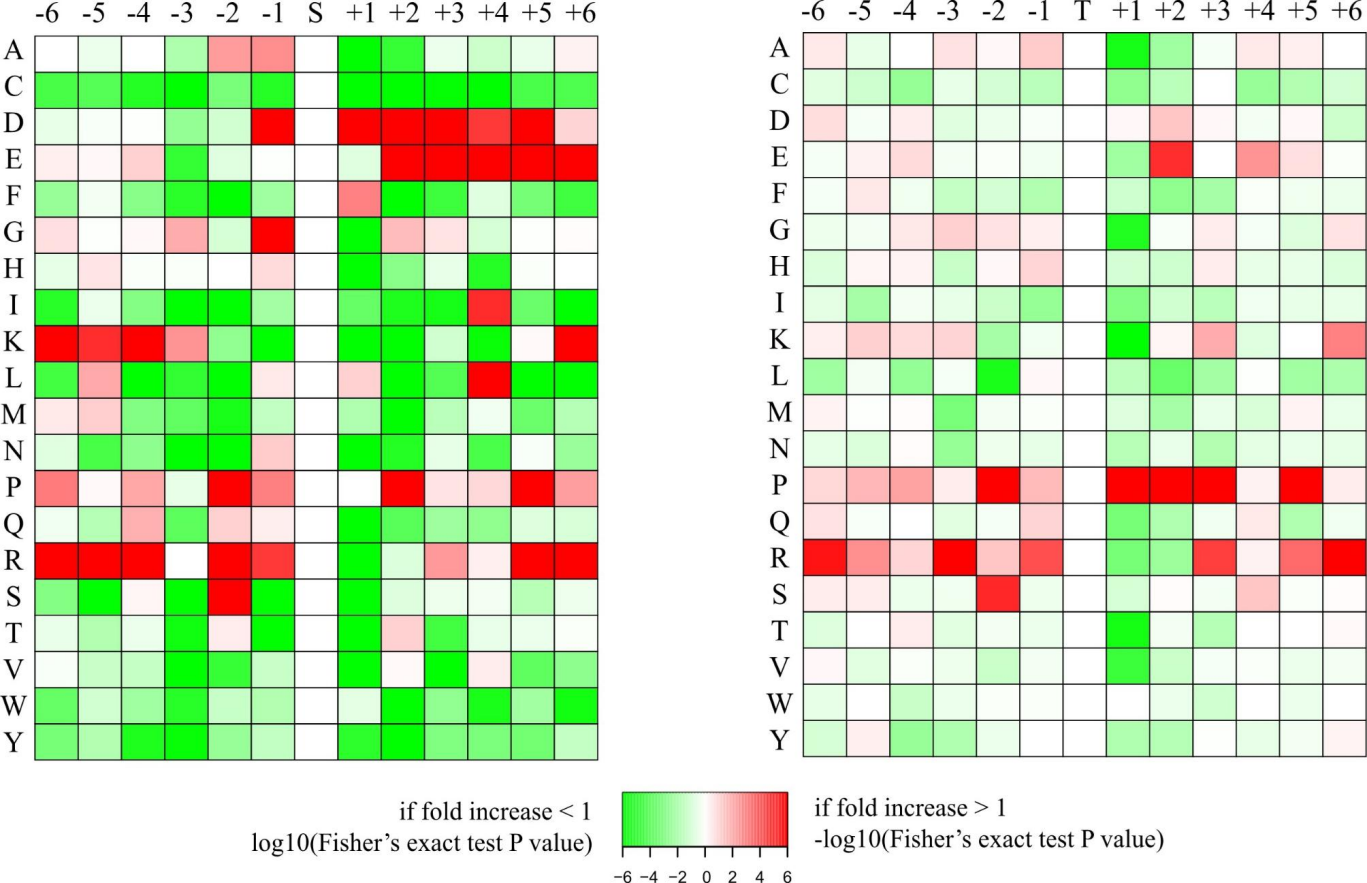
Heat maps of enrichment of six amino acid motifs upstream and downstream of serine (left) and threonine (right) modification sites. The letters on the left of each panel represent amino acids. Red indicates that this amino acid is significantly enriched near the modification site, and green indicates that the secondary amino acid is significantly reduced near the modification site

The specific amino acid (AA) sequence features or motifs around the phosphosites usually determine the kinase substrate specificity. In this study, Motif-X was used to detect the motifs around the identified phosphosites. Eighteen motifs were detected from phosphate groups (Fig. 3), including 17 pSer motifs and 1 pThr motifs (Table 1).These results suggest that acidic kinase may be the major kinase group involved in phosphorylation of the identified phosphoproteins during follicle selection in chicken.

**Table 1:** Motif enrichment analyzed with Motif-X

Motif	Motif Score	Motif Type
…R.SP….	32	14-3-3 mode
……SP…R.	32	
……SP.R…	32	CDK
……SP.K…	31.95	CDK
…G.SP….	25.78	
…RR.S……	32	PKA
….P.SP….	24.96	MAPK
……SP.S.	22.22	
…RS.S……	32	Art-like
……SP….	16	CDC2 kinase
…R.S……	16	CLK2 kinase
……SD.E…	32	CK2
……S.E….	16	G-CK
……S.D….	16	AMK2
……S.P….	16	
….R.S……	16	
….S.S……	7.9	
……TP….	16	

### Upregulation of LSD1Ser54p during chicken follicle selection

**Fig. 4:**
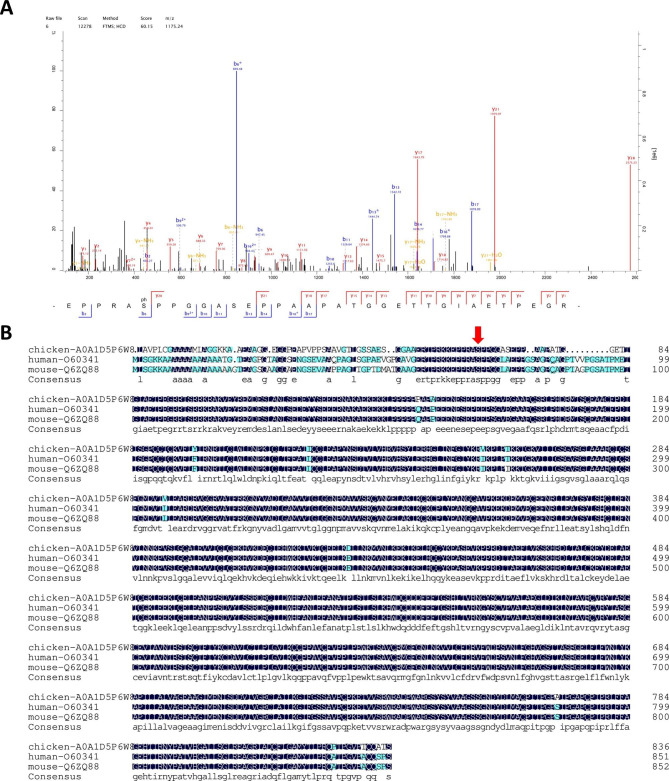
The MS2 spectrum of the chicken LSD1 protein (**A)** and amino acid sequence alignment of chicken, human and mouse LSD1 **(B)**. Arrow indicates the conserved amino acid of Ser at the 54^th^ site of chicken LSD1.

**Fig. 5:**
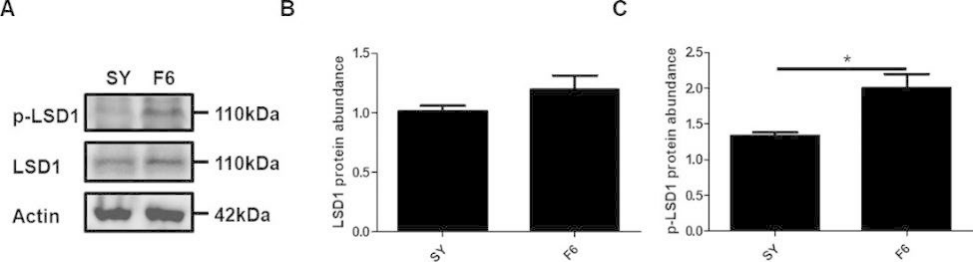
Expression of LSD1 protein (A, B) and LSD1Ser54p (A, C) in SY follicles and F6 follicles

Phosphoproteomic analysis indicated that there was significant difference in phosphorylation of serine at the 54th site of the LSD1 (LSD1Ser54p), but there was no significant difference in that of other sites **(**Fig. 4A**)**. Alignment of amino acid sequences of LSD1 protein of chicken, human and mouse revealed that the 54th Ser site was highly conserved **(**Fig. 4B**)**. The expression dynamics of chicken LSD1 protein and LSD1Ser54p during follicle selection (transition from SY follicle to F6 follicle) were examined, showing no significant difference of LSD1 protein expression (*p* > 0.05) **(**Fig. 5A and B**)**, but an upregulation of LSD1Ser54p from SY to F6 follicles (*p* < 0.05) **(**Fig. 5A C**)**. However, transcriptomic analysis indicated no significant difference of LSD1 expression at mRNA level between F6 follicles and SY follicles (*p* > 0.1, https://www.ncbi.nlm.nih.gov/sra/docs/, accession number SRP236909 and Supplementary Table 1).

### Localization and expression changes of LSD1Ser54p in follicular tissues and cells

**Fig. 6:**
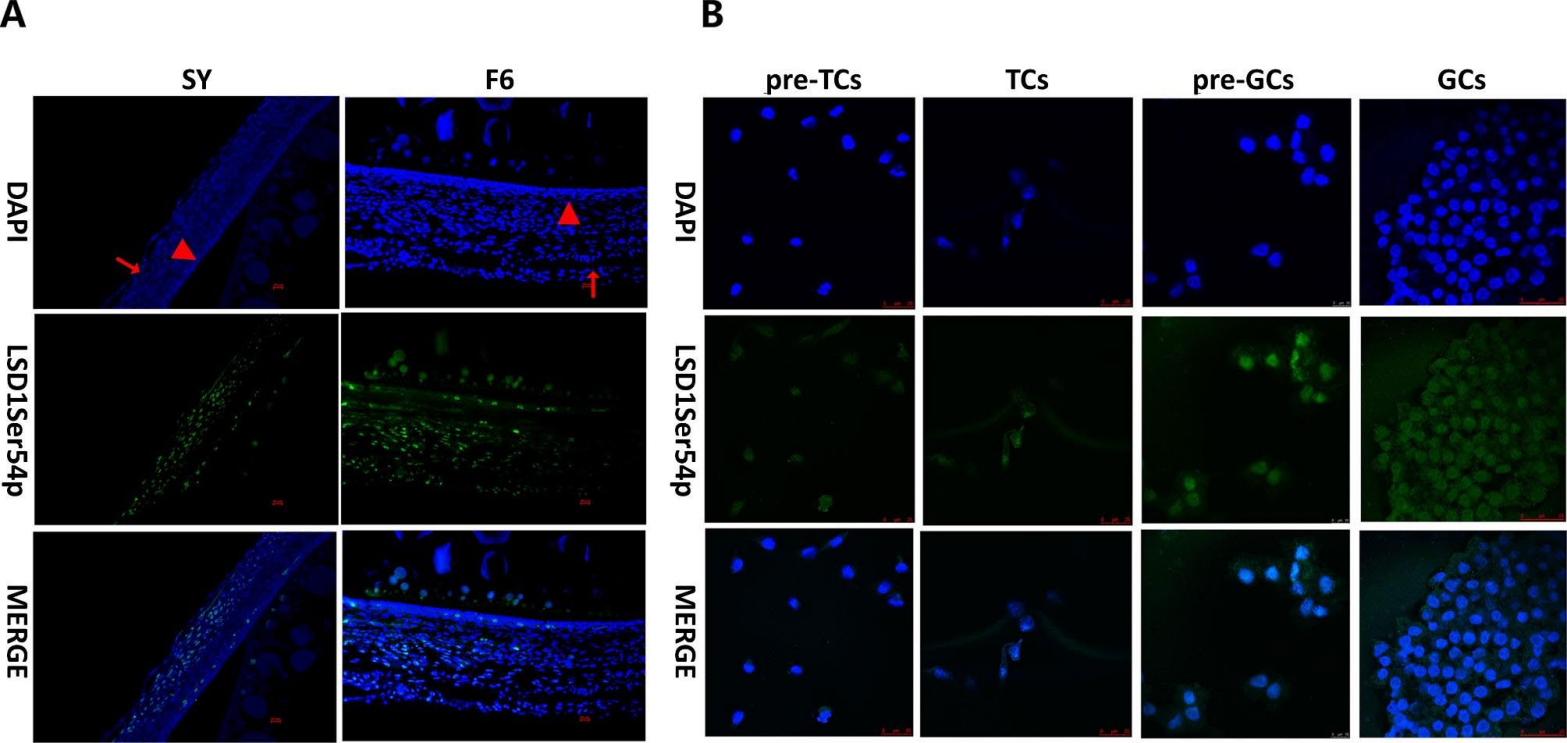
The localization of LSD1Ser54p in chicken SY and F6 follicles **(A)** and pre-TCs, TCs, pre-GCs and GCs cells **(B)**. The staining results were observed with a laser confocal microscope (Leica). Green, LSD1Ser54p; Blue, nucleus. Arrowhead, granulosa cells layer;Arrow, theca cells layer. Bar, 10 μm,25 μm

**Fig. 7:**
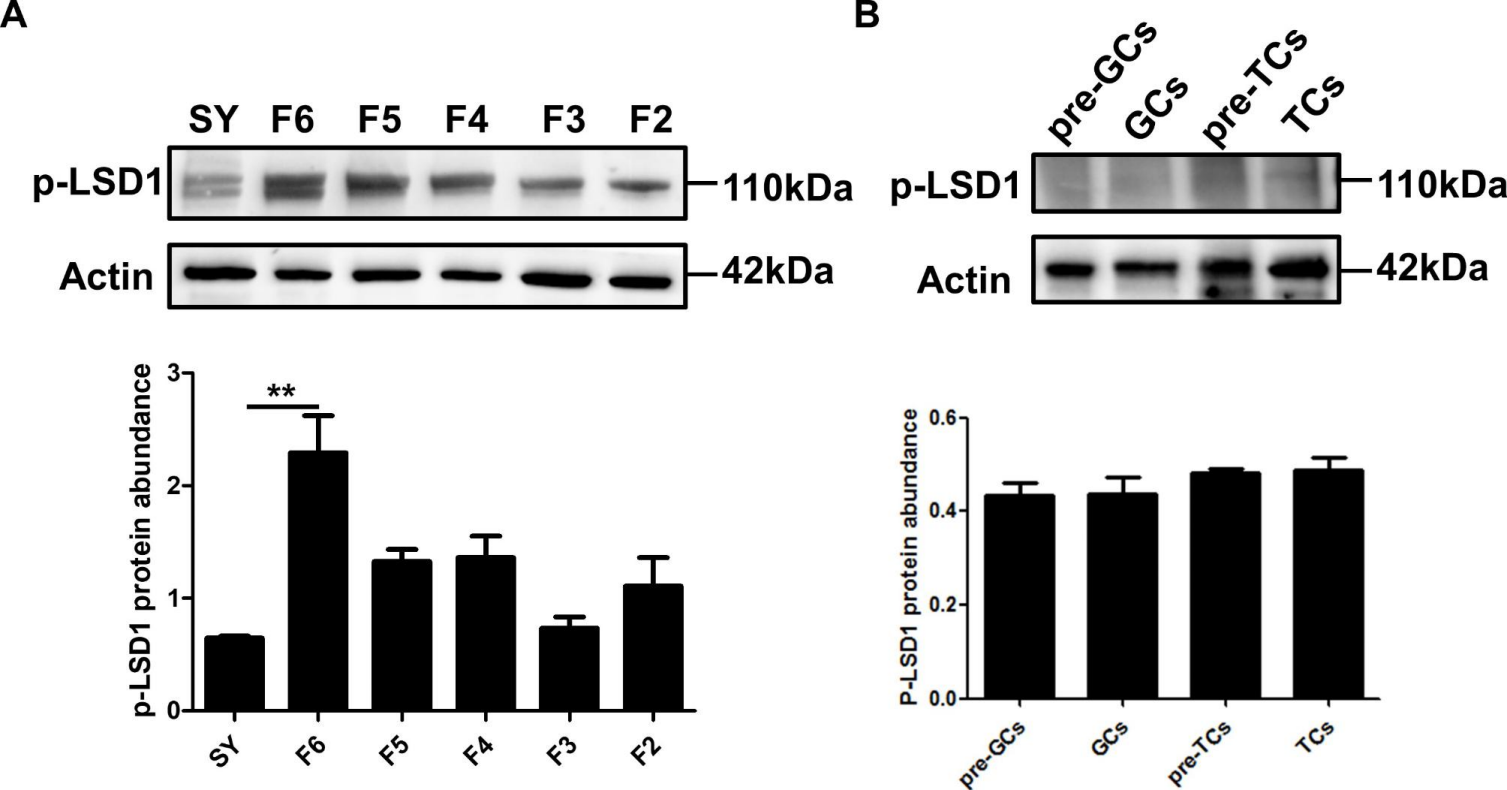
The expression level of LSD1Ser54p in follicular tissues and cells. (**A**) The expression changes of LSD1Ser54p in SY, F6, F5, F4, F3, F2 follicles. (**B**) The expression changes of LSD1Ser54p in Pre-GCs, GCs, pre-TCs and TCs. Data are presented as the mean ± SEM from at least three independent experiments. ^**^p < 0.01

The localization of LSD1Ser54p in SY and F6 follicles, as well as in pre-TCs, TCs, pre-GCs and GCs was conducted by immunofluorescence with customized specific antibody against chicken LSD1Ser54p. LSD1Ser65p is detected in both SY and F6 follicles, and was higher in F6 follicle than that of SY follicle **(**Fig. 6A**)**. LSD1Ser54p expression was mainly detected in the nucleus of pre-TCs, TCs, pre-GCs and GCs cells **(**Fig. 6B**)**. Western blotting analysis indicated that the expression of LSD1Ser54p was significantly increased in F6 follicles than in SY follicles (^**^p<0.01) **(**Fig. 7A**)**, which was consistent with the immunofluorescence results, while no significant changes were detected among pre-TCs, TCs, pre-GCs and GCs cells **(**Fig. 7B**)**.

### Effects of FSH on LSD1Ser54p level in chicken follicular cells

**Fig. 8:**
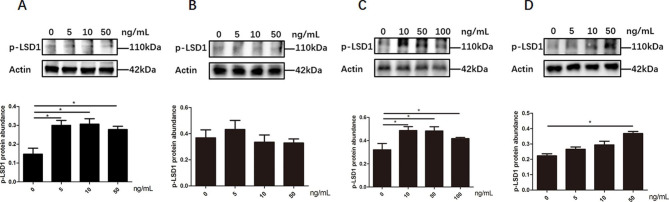
The expression level of LSD1Ser54p in pre-TCs**(A)**,TCs **(B)**, pre-GCs **(C) and** GCs **(D)** cells after treatment with different concentrations of FSH. ^*^p < 0.05

The effect of FSH on LSD1Ser54p level were different in four types of chicken follicular cells. In pre-TCs and pre-GCs, lower concentration of FSH treatment, at 5ng/mL **(**Fig. 8A**)** and 10ng/mL **(**Fig. 8C**)**, respectively, stimulated the expression of LSD1Ser54p (p < 0.05). In GCs, only at high concentration of 50 ng/mL FSH treatment significantly increased the expression of LSD1Ser54p (p < 0.05) **(**Fig. 8D**)**; however, in TCs, no effect of FSH on LSD1Ser54p was detected **(**Fig. 8B**)**.

### Effect of estradiol (E2) on LSD1Ser54p level in chicken follicular cells

**Fig. 9:**
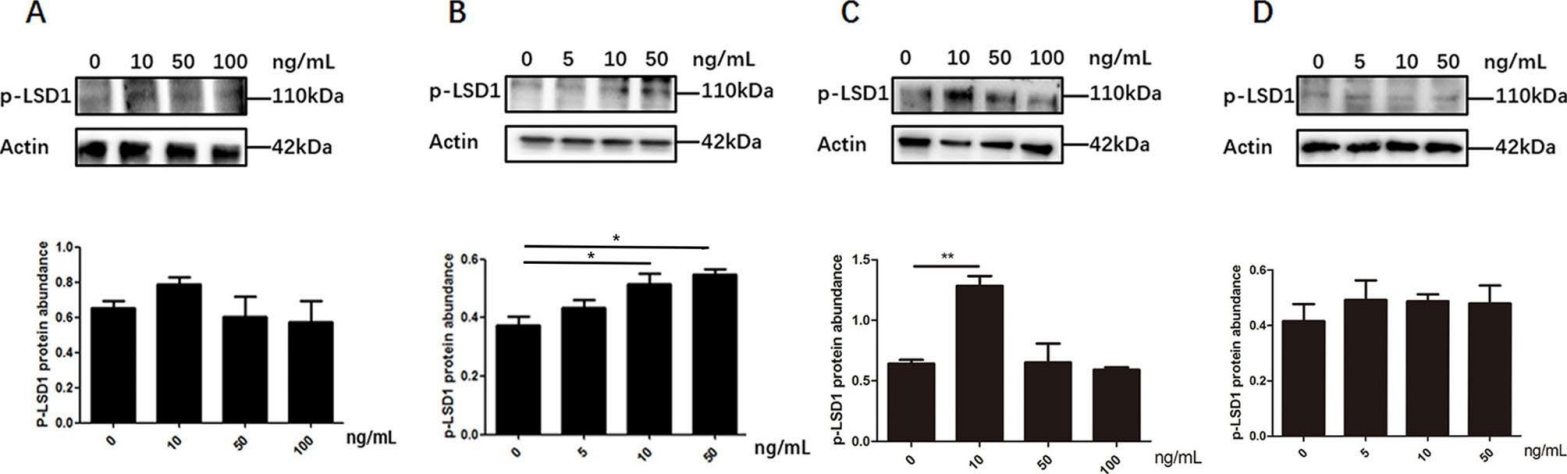
The expression level of LSD1Ser54p in pre-TCs **(A)**, TCs **(B)**, pre-GCs **(C) and** GCs **(D)** cells after treatment with different concentrations of estradiol (E2). ^*^p < 0.05, ^**^p < 0.01

In TCs, treatments with E2 at a concentration from 5 ng/mL to 50 ng/mL significantly increased the expression of LSD1Ser54p, exhibiting a dose effect **(**Fig. 9B**)**. In pre-GCs, 10 ng/mL of E2 increased the expression of LSD1Ser54p, but when the concentration of E2 was higher, its effect on LSD1Ser54p vanished **(**Fig. 9C**)**. E2 has no significant effect on the expression of LSD1Ser54p in pre-TCs **(**Fig. 9A**)** and GCs **(**Fig. 9D**)**.

### Effects of progesterone on LSD1Ser54p level in chicken follicular cells

**Fig. 10:**
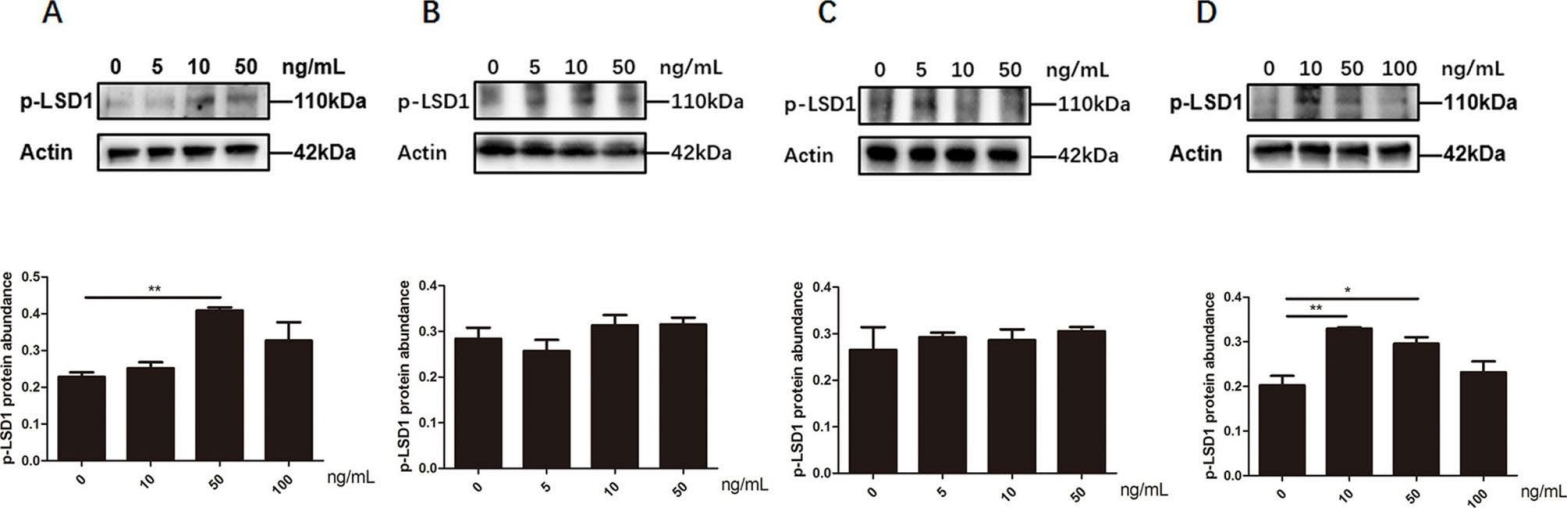
The expression level of LSD1Ser54p in pre-TCs **(A)**, TCs **(B)**, pre-GCs **(C) and** GCs **(D)** cells after treatment with different concentrations of progesterone. ^*^p < 0.05, ^**^p < 0.01

In pre-TCs, the expression level of LSD1Ser54p increased significantly after treatment with 50 ng/mL of progesterone (*p* < 0.05) **(**Fig. 10A**)**. In GCs, progesterone stimulated the expression level of LSD1Ser54p at 10 ng/mL (*p* < 0.01), however, the effect gradually decreased along with higher concentration **(**Fig. 10D**)**. No effect of progesterone was detected on the expression level of LSD1Ser54p in TCs **(**Fig. 10B**)** and pre-GCs **(**Fig. 10C**)**.

## Discussion

In chicken, follicle selection is regulated by a multiple of signal pathways, of which phosphorylation of proteins being involved in signal transduction plays vital roles. In this study, by phosphoproteomic analysis on SY and F6 follicles around follicle selection, we identified hundreds of differentially expressed phosphosites and further analyzed the dynamics and regulation of LSD1Ser54p in chicken pre-hierarchal and hierarchal follicles. For the first time, we found that the level of LSD1Ser54p was regulated by FSH, estrogen and progesterone, with effect being different *prior to* and *post* follicle selection and between theca and granulosa cells, suggesting an important role of LSD1Ser54p in chicken follicle selection.

In this study, about six thousand phosphosites were identified in chicken SY and F6 follicles, of which nearly two hundreds were significantly different between SY and F6 follicles, showing that the labeling technology with tandem mass labeling could be used for phosphorylated proteomics. Due to that follicle selection from SY to F6 is characterized with the differentiation of granulosa cells, the expression and regulation of LSD1Ser54p were subsequently examined. LSD1 is a histone lysine specific demethylase. As an important epigenetic erasure, LSD1 plays important roles in various physiological processes by regulating the transcription of essential genes. It can demethylate H3K4me2 into H3K4me1 or H3K4me0 when it forms a complex with CoREST transcription inhibition complex, demethylate H3K9me2 when forming a complex with androgen receptor [18, 19], and also remove the methylation of lysine in proteins other than histones by antagonizing the effect of Set7 / 9 [20]. The catalytic mechanism of LSD1 is to transfer hydride from methylated lysine to FAD cofactor, generate unstable imine intermediate, and release formaldehyde after hydrolysis [21]. The phosphorylation of LSD1 serine has important biological functions as reported in several studies. Phosphorylation of human LSD1 at 131^th^ and 137^th^ serine promotes the recruitment of LSD1 and ubiquitin p53 binding protein 1 at the DNA damage site, and contributes to DNA damage repair [22]. In breast cancer cells, phosphorylation of LSD1 at 112^th^ serine enhances the binding capacity and demethylation activity of LSD1 [23, 24], mutation from serine to alanine at this site exacerbates the pathogenesis of CSE/LPS-induced chronic obstructive pulmonary disease in mice [25] and inhibition of this phosphorylation alleviates colitis symptoms induced by dextran sulfate sodium [26]. However, phosphorylation of LSD1 54th serine was not reported in previous studies. In this study, for the first time, we characterized the expression dynamics and regulation of LSD1Ser54p by sex hormones during chicken follicle selection.

By both Western blotting and immunofluorescence analyses, we found that the level of LSD1Ser54p increased in F6 follicles compared to SY follicles. The phosphorylation of LSD1 at 54th serine was found both in theca and granulosa cells, with no significant difference of LSD1Ser54p level between them neither in pre-hierarchal nor hierarchal chicken follicles, although a little higher in theca cells. The distribution of LSD1Ser54p is mainly found in the nucleus, which is consistent with its demethylation activity on histone. Differences in the effect of sex hormones on LSD1Ser54p were found among pre-TCs, TCs, pre-GCs and GCs cells. No obvious effect on LSD1Ser54p was found of E2 in pre-TCs and GCs, and of P4 in TCs and pre-GCs. Follicle selection in chicken is characterized by the differentiation of granulosa cells and the expression of steroidogenic acute regulatory protein (*StAR*) and cytochrome P450 family 11 subfamily A member 1 (*CYP11A1*) that is prerequisite for progesterone synthesis [27]. We found that, FSH and progesterone treatment elevated the level of LSD1Ser54p in GCs, which is consistent with the responsiveness of chicken hierarchal follicles to FSH and progesterone [7]. The proliferation of theca cells is another feature of follicle selection. The stimulation of LSD1Ser54p in pre-TCs by progesterone, and in TCs by FSH and E2, suggesting that LSD1 might also involve in the function of theca cells in chicken follicles. The effects of FSH and E2 on the level were different in GCs and TCs (Figs. 8 and 9), suggesting that LSD1Ser54p level was differentially regulated by FSH and E2. This difference is likely caused by the different function of GC and TC in the growing stage of hierarcal follicles, which requires further investigations.

In this study, we verified the changes of LSD1Ser54p before and after follicle selection, the subcellular localization and expression in four types of chicken follicular cells, and its regulation by FSH, estrogen, and progesterone. It is speculated that the significant up-regulation of LSD1Ser54p after follicle selection enhances the function of LSD1 demethylation, thereby inhibiting or promoting the expression of follicle selection-related genes and promoting the occurrence of follicle selection. The regulation of LSD1Ser54p by hormones predicts that LSD1Ser54p participates in the opening or closing of hormone-dependent pathways or genes, such as the TGF-β pathway, which controls the development of follicles through demethylation in the pathway, but the specific mechanism of action needs further study.

Apart from LSD1Ser54p, the function of other significantly differentially expressed phosphorylated proteins in chicken follicle selection needs to be further investigated in future studies. Understanding the function of these differentially expressed phosphorylated proteins will expand our knowledge of how phosphorylation act to regulate follicle selection, and provide references for elucidating mechanisms underlying follicle development in mammals including human.

## Conclusion

Phosphoproteomic analysis on chicken ovarian follicles around follicle selection identified hundreds of differentially expressed proteins, among which LSD1Ser54p level increased from 6 to 8 mm small yellow follicles to F6 follicles and was upregulated by FSH, estrogen progesterone in different ways in chicken follicular cells, suggesting that LSD1Ser54p is involved in chicken follicle selection.

## Methods

### Tissue collection

Tissues of small yellow follicles and hierarchical follicles 12–15 mm in diameter used for phosphoproteomic analysis were described in reference (11). Briefly, 15 Hy-Line brown hens randomly sampled from the same batch were divided into three biological groups (for each group, n = 5), which had been laying regularly for at least one month (28 weeks old, with a mean body weight of 2.1 ± 0.12 kg) and were housed under standard conditions with free access to food and water. Vaccination was performed according to the recommendations from Hy-Line International. For sampled hens, the laying time was recorded and were killed by cervical dislocation approximately ten hours after laying. From each hen, small yellow follicles 6–8 mm in diameter (designated as SY ) and hierarchal follicles 12–15 mm in diameter (designated as F6) were separately collected, and the egg yolk was carefully squeezed out with tweezers, washed with phosphate-buffered saline (Thermo Fischer Scientific, MA, USA), immediately frozen in liquid nitrogen and used for phosphorproteomic analysis. Totally three biological replicates were prepared for the phosphoproteomic analyses of total proteins.

### Protein extraction, digestion, and TMT labeling

Protein extraction, digestion and TMT labeling from SY and F6 follicles were carried out according to reference (11). Briefly, lysis buffer containing 8 M urea (Sigma Aldrich, MO, USA) and 1% protease inhibitor cocktail (Merck Millipore, MA, USA) was used to extract proteins from the tissues, the concentration of which was determined with a BCA kit (Beyotime, Shanghai, China) according to the manufacturer’s instructions. Then, protein enzymolysis was performed using trypsin (Promega, WI, USA), the peptides obtained were desalted on a Strata X C18 SPE column (Phenomenex, CA, USA) and vacuum-dried. Finally, the peptides were reconstituted in 0.5 M TEAB and processed according to the manufacturer’s protocol for the TMT kit (Thermo Fischer Scientific, MA, USA).

### Phosphopetide enrichment

Phosphopeptides were enriched with TiO2-based phosphopeptide enrichment as described by Larsen et al. with some modifications [28]. Briefly, Peptide mixtures were first incubated with IMAC microspheres suspension with vibration in loading buffer (50% acetonitrile/6% trifluoroacetic acid). The IMAC microspheres with enriched phosphopeptides were collected by centrifugation, and the supernatant was removed. To remove nonspecifically adsorbed peptides, the IMAC microspheres were washed sequentially with 50% acetonitrile/6% trifluoroacetic acid and 30% acetonitrile/0.1% trifluoroacetic acid. To elute the enriched phosphopeptides from the IMAC microspheres, elution buffer containing 10% NH_4_OH was added and the enriched phosphopeptides were eluted with vibration. The supernatant containing phosphopeptides was collected and lyophilized for LC-MS/MS analysis.

### Liquid chromatography coupled with tandem mass spectrometry (LC-MS/MS)

LC-MS/MS was performed according to reference (11). The tryptic peptides were fractionated into fractions by high pH reverse-phase HPLC using Thermo Betasil C18 column (5 μm particles, 10 mm ID, 250 mm length), dissolved in 0.1% formic acid (solvent A), directly loaded onto a home-made reversed-phase analytical column (15 cm length, 75 μm i.d.). The gradient was comprised of an increase from 6 to 23% solvent B (0.1% formic acid in 98% acetonitrile) over 26 min, 23–35% in 8 min and climbing to 80% in 3 min then holding at 80% for the last 3 min, all at a constant flow rate of 400 nL/min on an EASY-nLC 1000 UPLC system.

The resulting MS/MS data were processed using the Maxquant search engine (v.1.5.2.8) as in reference 11. The tandem mass spectra were searched against the *Gallus gallus* database (http://www.uniprot.org/proteomes/UP000000539) concatenated with the reverse decoy database. The GO phosphoproteome was derived from the UniProt-GOA database (www.http://www.ebi.ac.uk/GOA/). The Kyoto Encyclopedia of Genes and Genomes (KEGG) database was used to identify the enriched pathways. The Soft motif-x (https://motif-x.med.harvard.edu/motif-x.html) was used to analyze the model of sequences constituted with amino acids in specific positions of modify-21-mers (10 amino acids upstream and downstream of the site) in all protein sequences. Phosphoproteins with a threshold of *p* < 0.05 and a fold change of > 1.5 or < 1/1.5 were identified as differentially expressed proteins (DEPs) between SY and F6 follicles. The MS phosphoproteomics data have been deposited in the ProteomeXchange Consortium via the PRIDE partner repository (https://www.ebi.ac.uk/pride/) with the dataset identifier PXD029503.

### Preparation and culture of theca and granulosa cells and hormone treatment

Preparation and culture of theca and granulosa cells were performed according to reference (11). Briefly, after careful removal of yolk from pre-hierarchal follicles that was washed with sterile PBS (Hyclone) with ophthalmic forceps, the follicles were digested with 0.1% collagenase II (MP Biomedicals, Santa Ana, CA, USA) at 38 °C for 15 min to obtain granulosa cells (designated as pre-GCs), for additional 30 min to obtain the theca cells (designated as pre-TCs). For hierarchal follicles, after the removal of yolk, granulosa cells (designated as GCs) were firstly separated from the theca externa cells with ophthalmic forceps, then digested with 0.25% trypsin-EDTA (Gibco-BRL, NY, USA) at 38 °C for 15 min, while the theca cells (designated as TCs) were obtained by digesting the theca externa and interna with 0.1% collagenase II at 38 °C for 30 min. After centrifugation, the pre-TCs, TCs, pre-GCs and GCs cells were separately suspended in culture medium containing M199 (Gibco-BRL, NY, USA), 10% fetal bovine serum (Biological Industries, Kibbutz Beit Haemek, Israel) and 1% penicillin/streptomycin (Solarbio, Beijing, China) and subsequently seeded at a density of 1 × 10 ^5^ cells/ well in 24-well culture plates and cultured at 38 °C in a water-saturated atmosphere of 95% air and 5% CO_2_. The number of viable cells was estimated using Trypan blue staining. After 24 h, cultured theca and granulosa cells were treated with different concentrations (0, 5, 10, and 50 ng/mL or 0, 10, 50, and 100 ng/mL) of recombinant FSH (R&D Systems, MN, USA), E2 (Sigma, MO, USA) and P4 (Sigma, MO, USA). All the treated cells were collected after another 24 h for protein extraction and Western blotting.

### Immunofluorescence

The SY and F6 follicles obtained from Hy-Line laying hens were washed three times with PBS (Phosphate Buffered Saline, HyClone), fixed with 4% paraformaldehyde (biosharp) for 20 min and paraffin-embedded, and then cut into 5 μm tissue sections that were deparaffinized before immunofluorescence. The follicular theca and granulosa cells were inoculated on the cell slides, cultured for 24 h and washed with PBS for three times, then fixed with 4% paraformaldehyde for 20 min, and washed with PBS for three times. For immunofluorescence observation, 0.2% TritionX-100 (Blotopped) was added to the tissue and cell slide prepared above, placed at 4 °C for 15 min, and then washed three times with PBS. The tissues and cells were sealed with 10% blocking serum (Normal Goat Serum, Solarbio) and kept at room temperature for 30 min. Rabbit customized LSD1Ser54p primary antibody(1:500) (AB clonal Technology) was added, treated at 37 °C for 1 h, and washed with PBS for three times. Secondary antibody (FITC-labeled Goat Anti-Rabbit lgG(H + L)) was added, protected from light at 37 °C for 1 h, and repeated washing with PBS for three times. DAPI (Beyotime) was added and the nuclei were stained for 3–5 min at room temperature and in the dark. After washing for three times, the slides were observed with fluorescence microscope (Leica).

### Western blotting

Phosphatase inhibitors and protease inhibitors were added into cell lysates (Cell lysis buffer for Western and IP, Beyotime) at a ratio of 1:50 to lyse follicle cells treated with different concentrations of hormones. Proteins were obtained by centrifugation at 12,000 rpm, and 4 °C for 5 min. Protein concentration was determined by the bicinchoninic acid assay (BCA Protein Array kit, TIANGEN Biotech). An equal amount of protein was separated by running on 4–15% SDS gel (BeyoGel™ Plus Precast PAGE Gel for Tris-Gly System) electrophoresis under denaturing and nonreducing conditions and then transferred to nitrocellulose filter membrane (PVDF). At room temperature, the membrane was sealed with confining liquid (NcmBlot Rapid Transfer Buffer) for 10 min and then incubated with rabbit customized LSD1Ser54p primary antibody (1:1000) in a 5% bovine serum albumin/PBS solution for 2 h at room temperature. After washing in PBST (G-Biosciences), the membranes were incubated with horseradish peroxidase-conjugated goat anti-rabbit secondary antibodies (1:1000; Beyotime) in a 5% bovine serum albumin/PBS solution for 2 h at room temperature and washed with PBST (Coolaber). The membrane is dipped into the luminescent solution (BeyoECLPlus A:B = 1:1) and developed with a C300 developer. The obtained protein bands were analyzed by the Image software, and the expression level of the target protein under different treatments was determined according to the gray value.

### Statistics

All Western blotting experiments were repeated at least three times and all data were presented as mean ± SEM. Student’s *t*-test was used to compare the LSD1 and LSD1Ser54p levels between SY and F6 follicles. For the comparison between different follicles or follicular cells treated with sex hormones, one-way ANOVA followed by Duncan’s multiple range test was employed for statistical analysis (SPSS 19.0, SPSS Inc., Chicago, IL). *p* < 0.05 was considered as significantly different.

## Electronic supplementary material

Below is the link to the electronic supplementary material.

**Additional File 1: Supplementary table 1**. Comparison of the expression level of chicken LSD1 mRNA in small yellow follicles and F6 follicles by RNA-seq.

**Additional File 2:** Image of original protein blotting.

## Data Availability

The reference proteome (http://www.uniprot.org/proteomes/ UP000000539, chicken proteome ID: UP000000539) database of the *Gallus gallus* were used in this study. The MS proteomics data have been deposited in the ProteomeXchange Consortium via the PRIDE partner repository (https://www.ebi.ac.uk/pride/) with the dataset identifier PXD029503.

## Abbreviations

BP: Biological process
CC: Cellular component
DEPs: Differentially expressed proteins
GO: Gene ontology
KEGG: Kyoto Encyclopedia of Genes and Genomes
GCs: granulosa cells
TCs: thecal cells.
