# Supplementary material for: Phosphoproteomic analysis on ovarian follicles reveals the involvement of LSD1 phosphorylation in Chicken follicle selection
Source: BMC Genomics. 2023 Mar 13;24:109. doi: 10.1186/s12864-023-09223-6 (PMC10012441; doi:10.1186/s12864-023-09223-6)
Supplement: Supplementary file 1 — Additional File 1: Supplementary table 1. Comparison of the expression level of chicken LSD1 mRNA in small yellow follicles and F6 follicles by RNA-seq. [file 12864_2023_9223_MOESM1_ESM.docx]

Supplementary table 1 Comparison of the expression level of chicken LSD1 mRNA in small yellow follicles and F6 follicles by RNA-seq

| Item | SY-1 | SY-2 | SY-3 | F6-1 | F6-2 | F6-3 |
| --- | --- | --- | --- | --- | --- | --- |
| Count | 3919 | 4075 | 4197 | 3775 | 4167 | 5115 |
| FPKM | 29.22478 | 28.85509 | 28.05430 | 28.00591 | 28.27914 | 29.67253 |
| Log_2_ foldchange | -0.002855556 | | | | | |
| *p*-value | 0.981028681 | | | | | |
| padj | 0.992937811 | | | | | |

Note: SY, small yellow follicles; F6, F6 follicles; FPKM, fragments per kilobase per million mapped fragments; padj, adjusted *p* value.
