# Supplementary material for: Phosphoproteomic analysis on ovarian follicles reveals the involvement of LSD1 phosphorylation in Chicken follicle selection
Source: BMC Genomics. 2023 Mar 13;24:109. doi: 10.1186/s12864-023-09223-6 (PMC10012441; doi:10.1186/s12864-023-09223-6)
Supplement: Supplementary file 2 — Additional File 2: Image of original protein blotting. [file 12864_2023_9223_MOESM2_ESM.pdf]

Image of original protein blotting

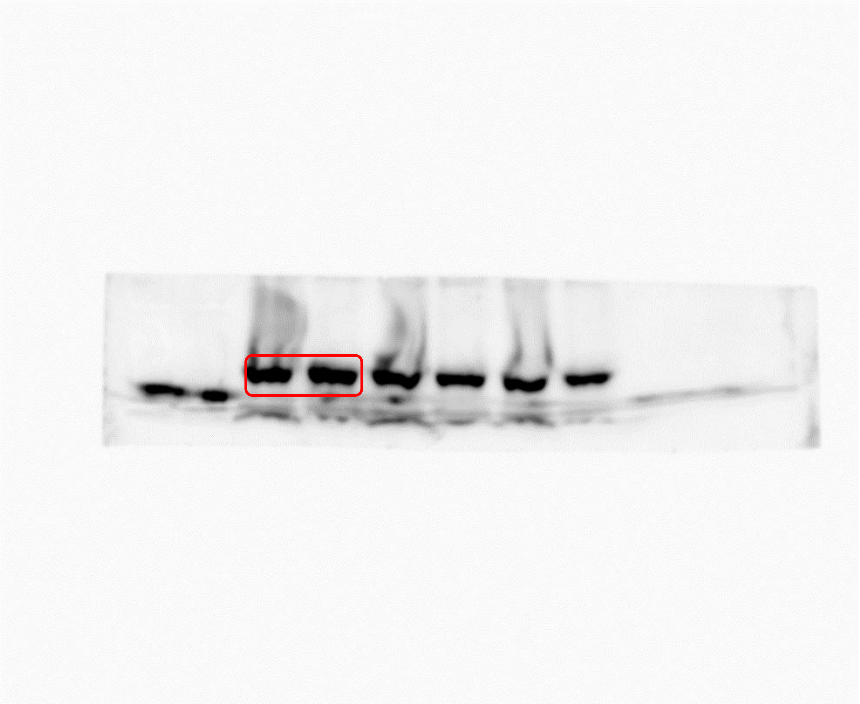

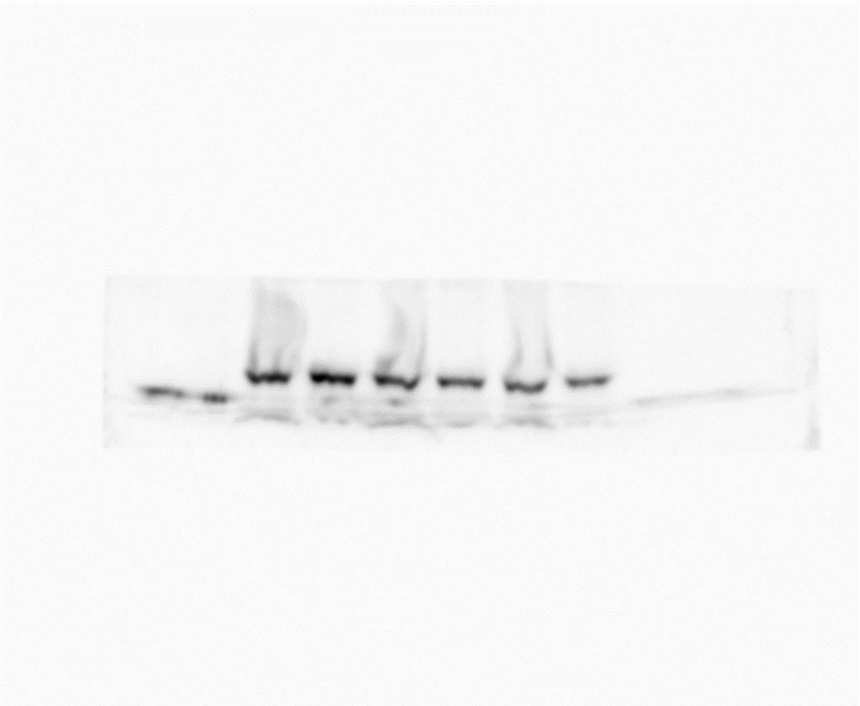

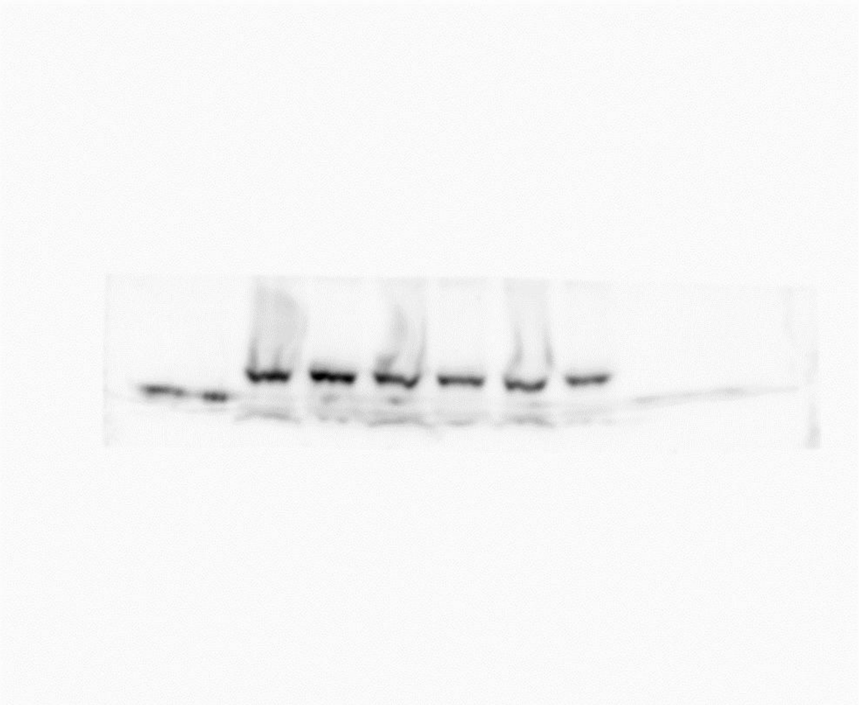

Figure 5A Original actin gel blotting image. There were three repeated blots in sequence and multiple exposure images. The images used in the manuscript are in the red boxes.

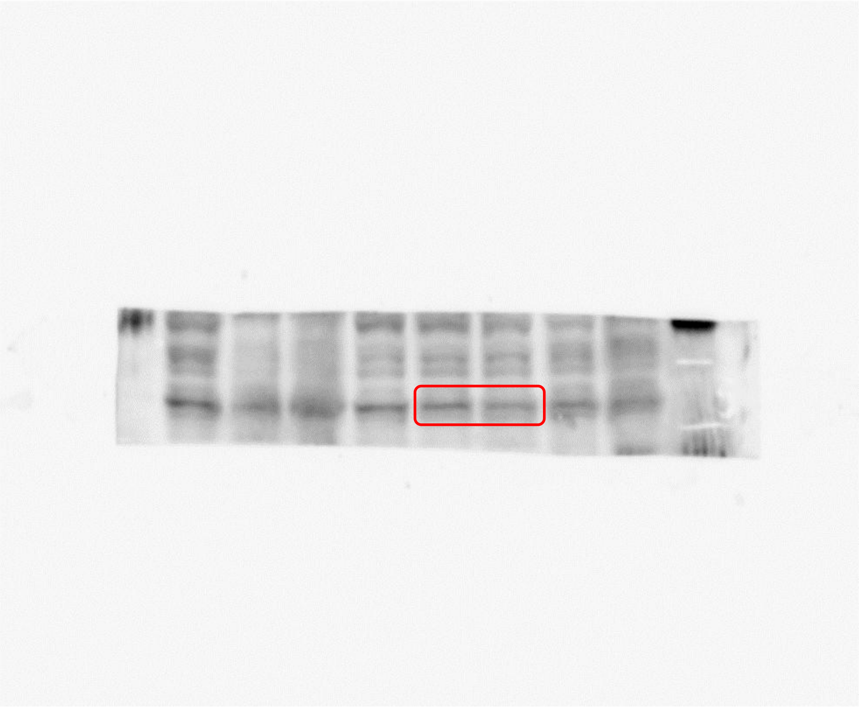

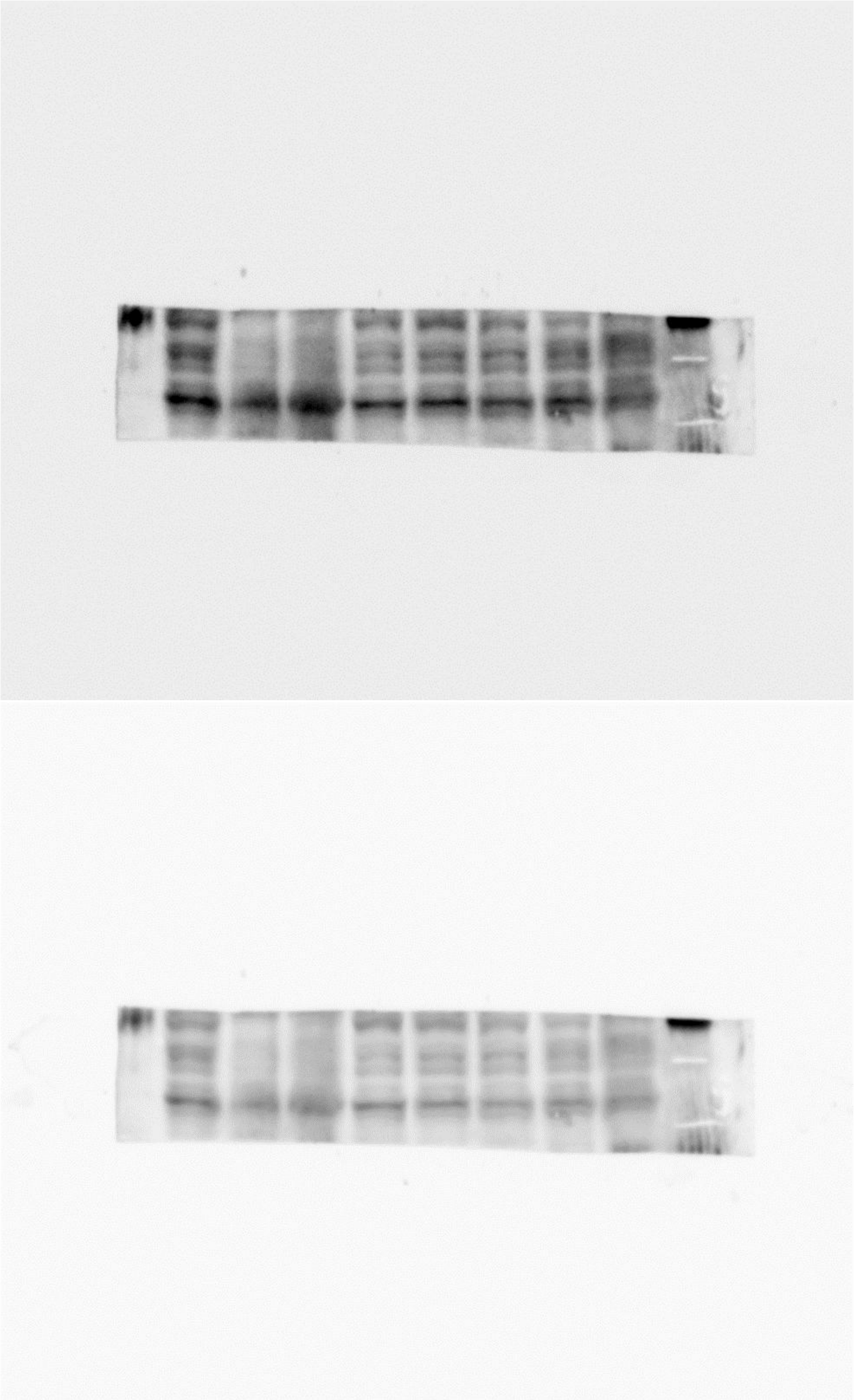

Figure 5A Original LSD1 gel blotting image .There were three repeated blots in sequence and multiple exposure images. The images used in the manuscript are in the red boxes.

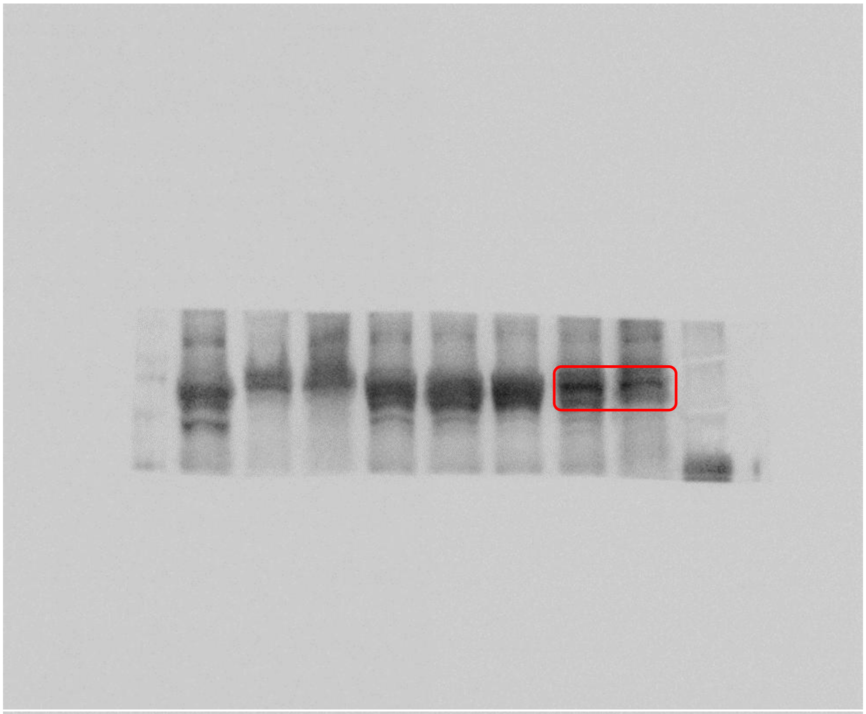

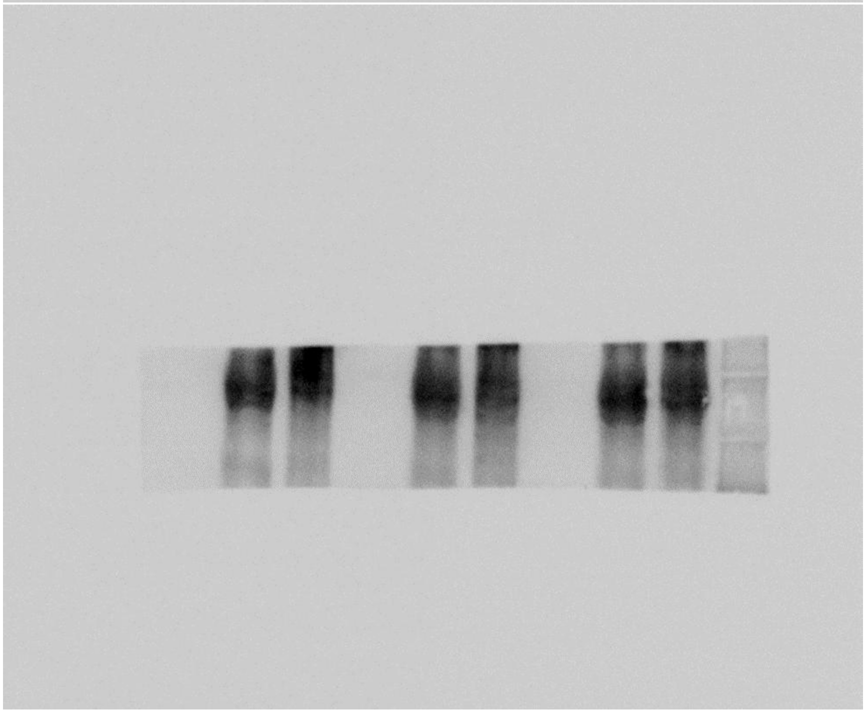

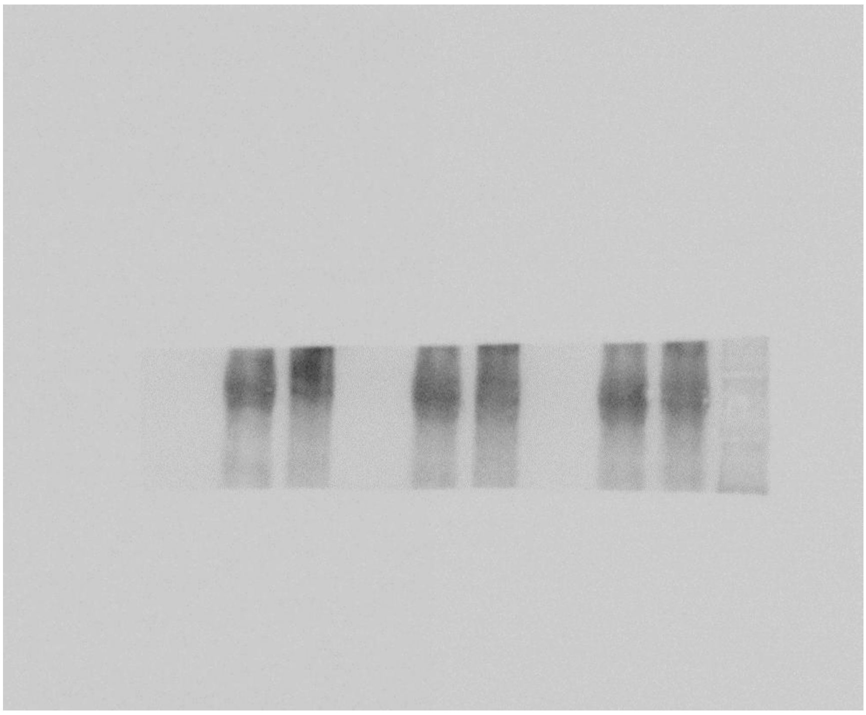

Figure 5A Original p-LSD1 gel blotting image and all replication. There were three repeated blots in sequence and multiple exposure images. The images used in the manuscript are in the red boxes.

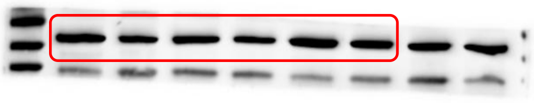

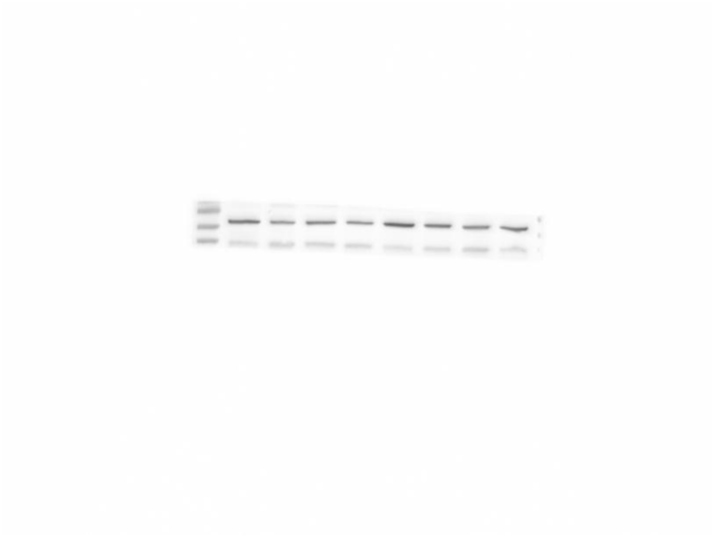

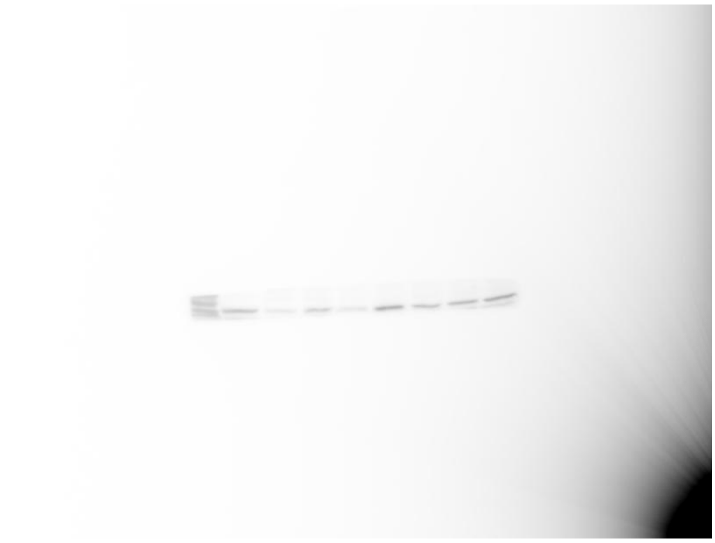

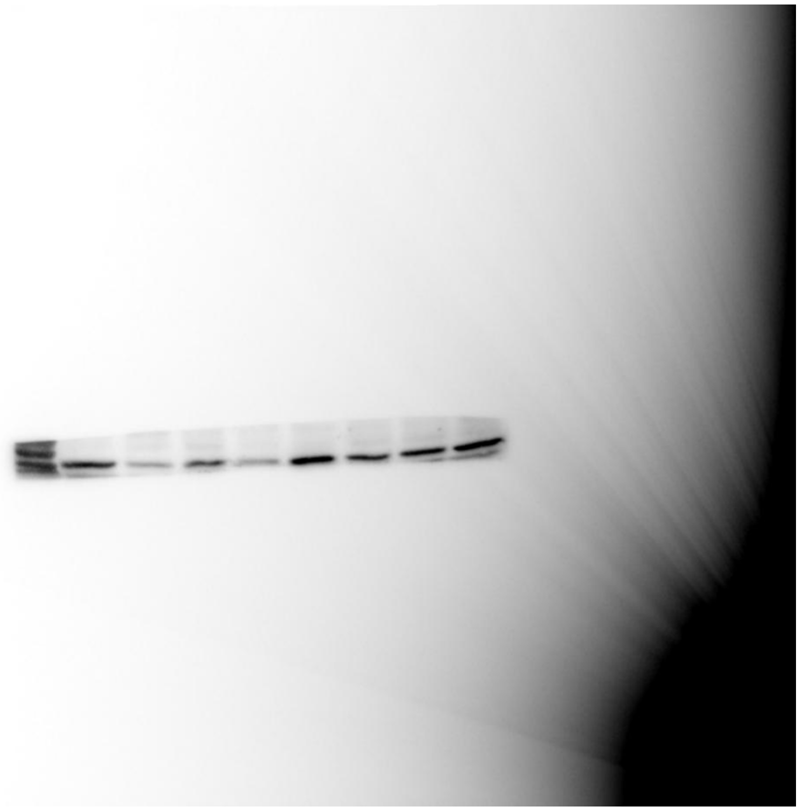

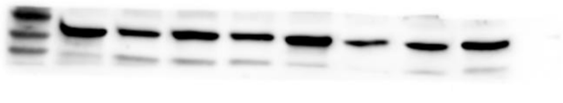

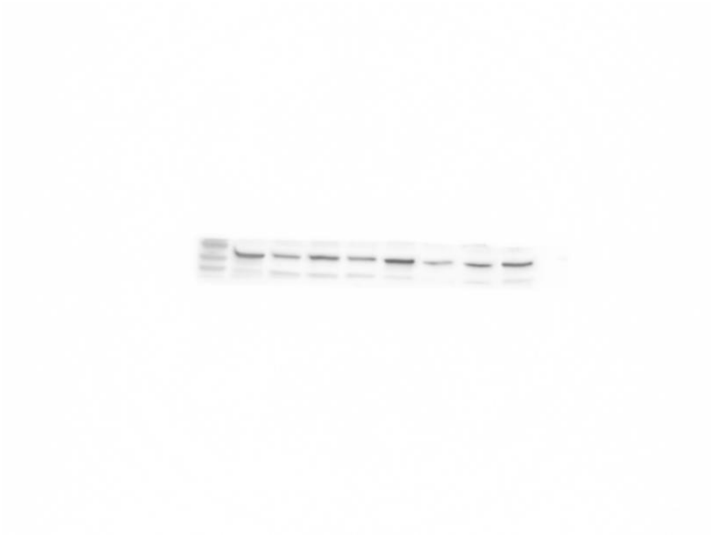

Figure 7A Original actin gel blotting image. There were three repeated blots in sequence and multiple exposure images. The images used in the manuscript are in the red boxes.

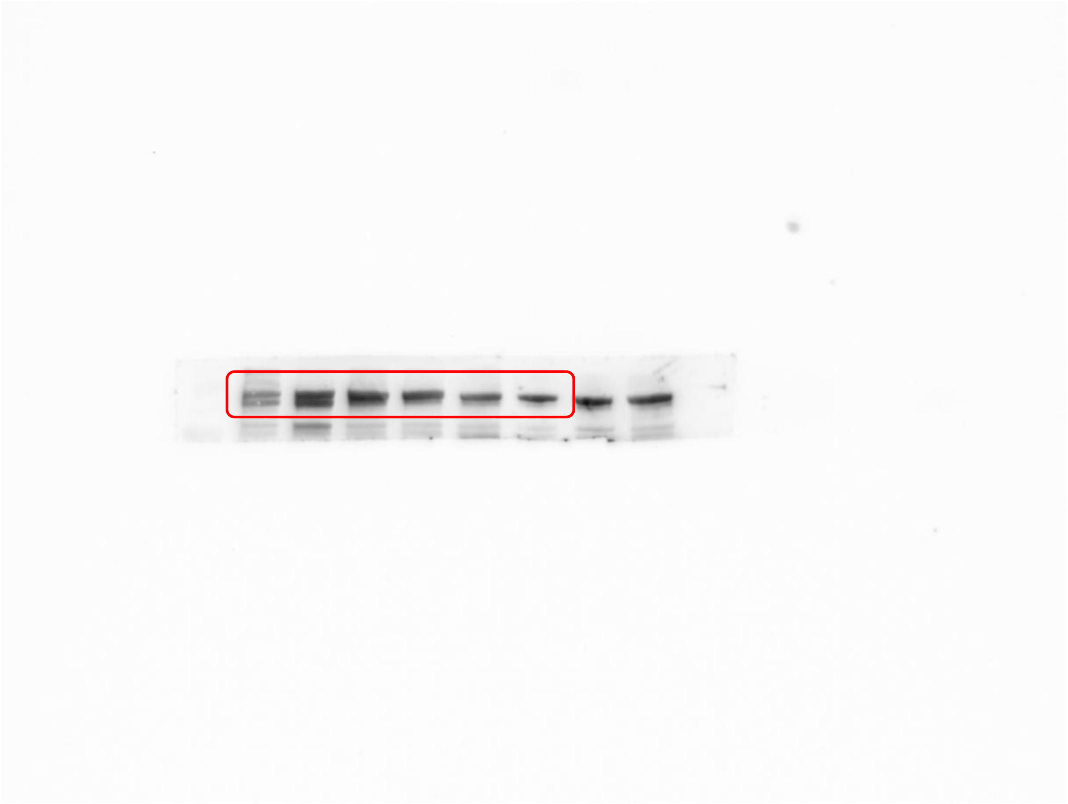

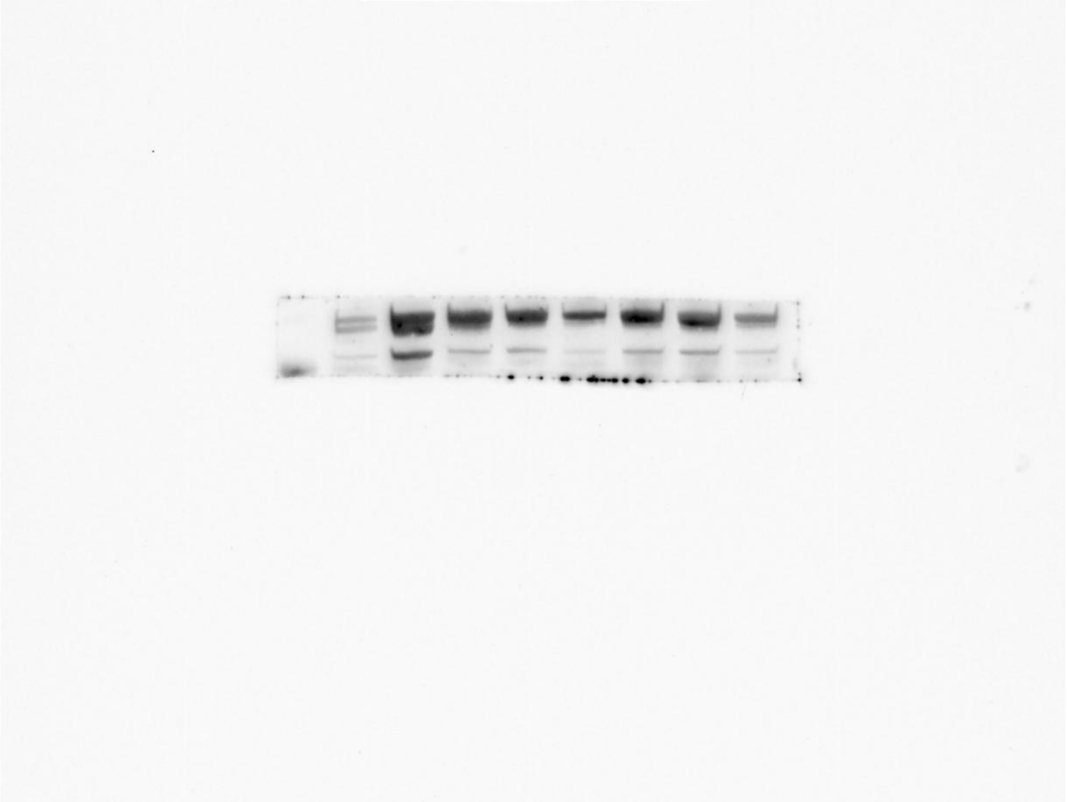

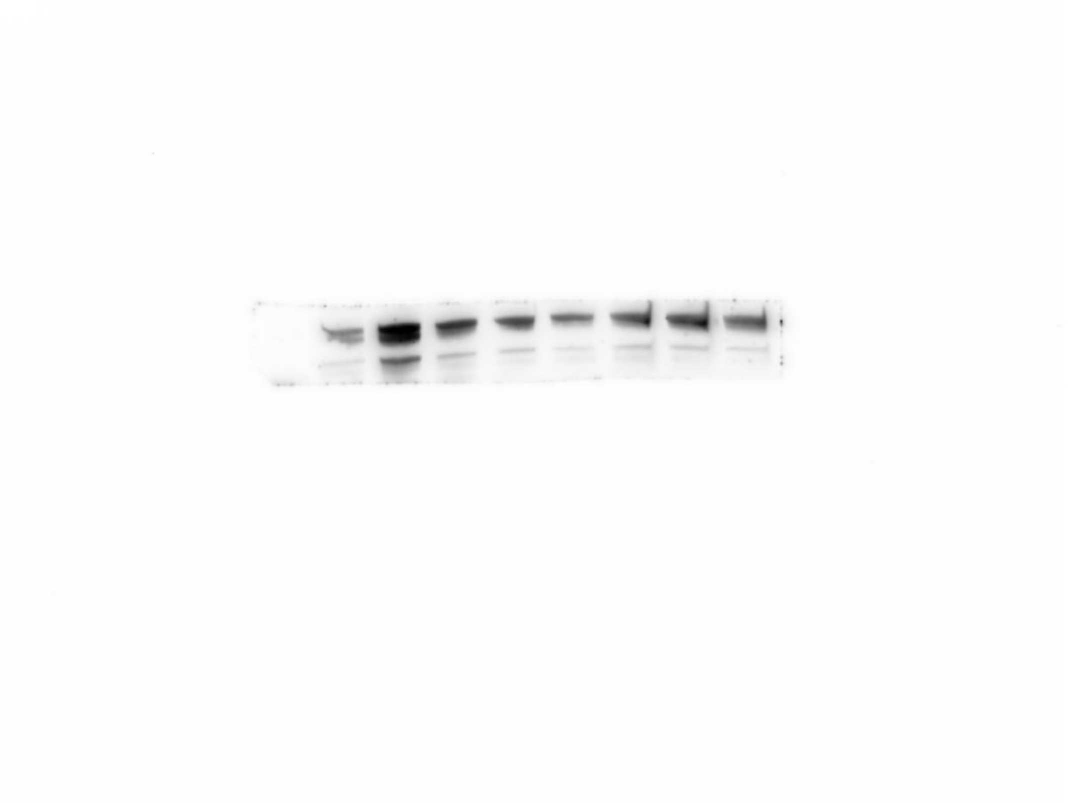

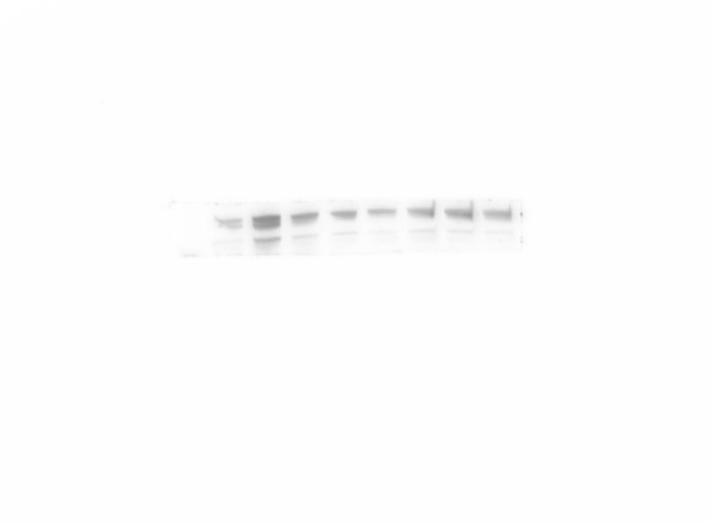

Figure 7A Original p-LSD1 gel blotting image. There were three repeated blots in sequence and multiple exposure images. The images used in the manuscript are in the red boxes.

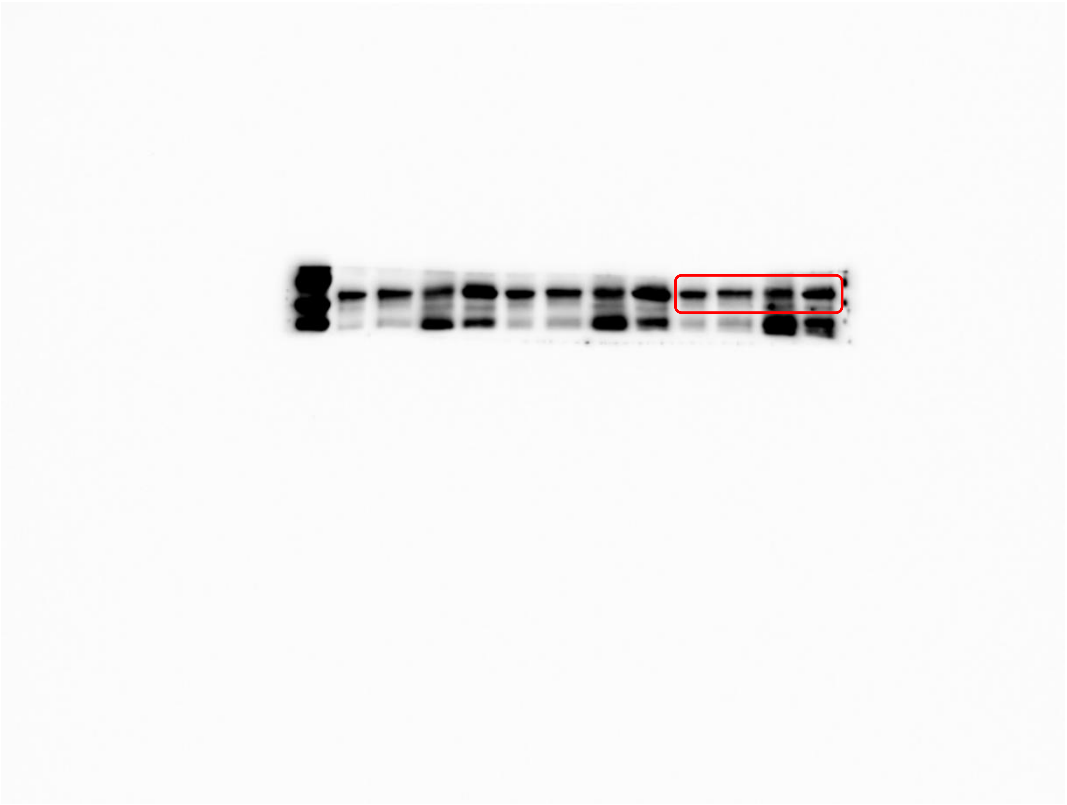

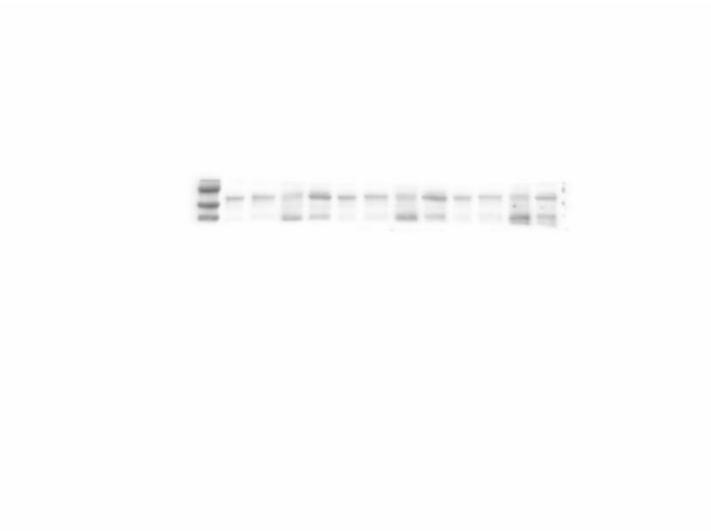

Figure 7B Original actin gel blotting image. There were three repeated blots in sequence and multiple exposure images. The images used in the manuscript are in the red boxes.

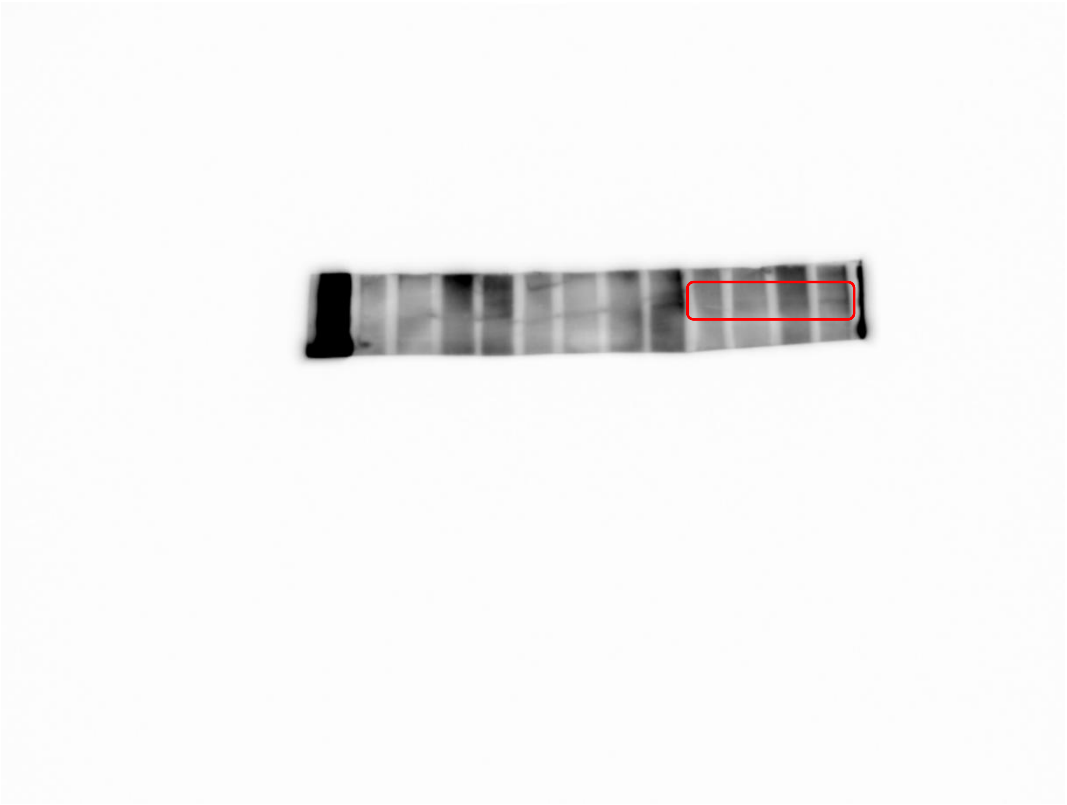

Figure 7B Original p-LSD1 gel blotting image and all replication， the images used in the manuscript are in the red boxes.

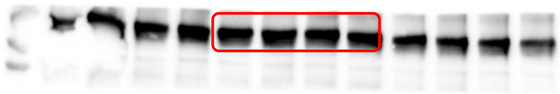

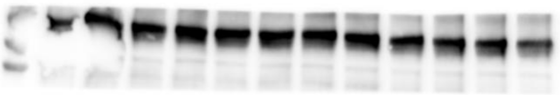

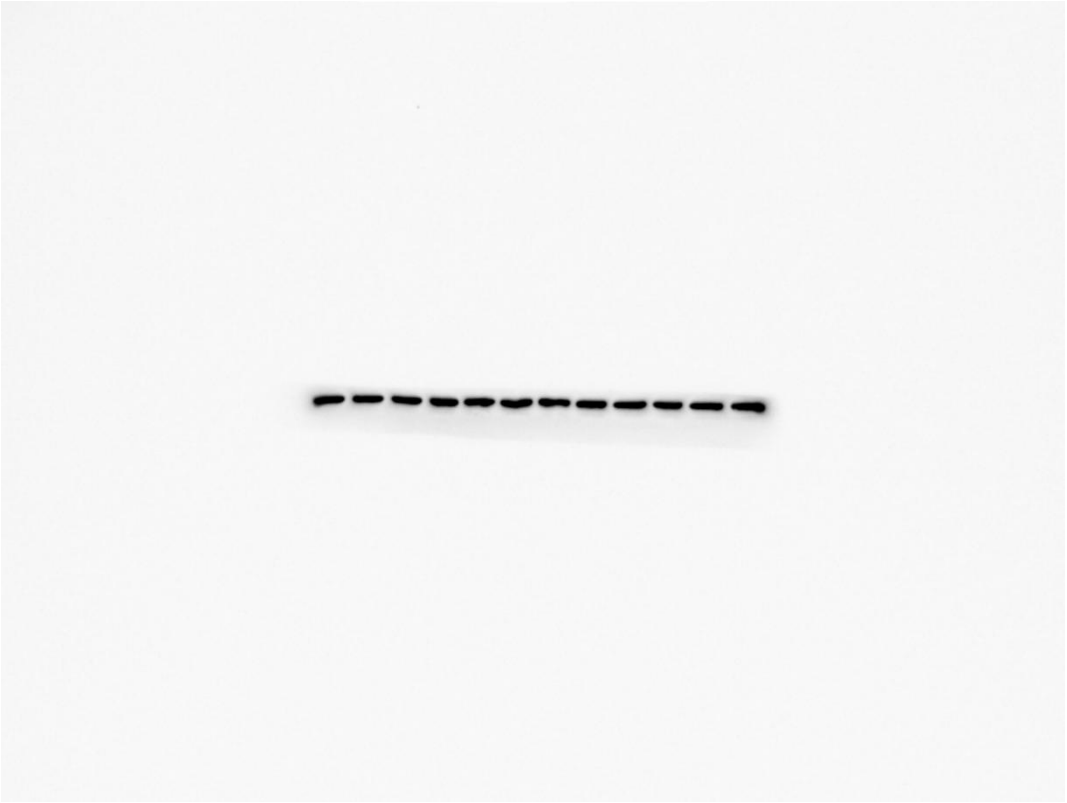

Figure 8A Original actin gel blotting image. There were three repeated blots in sequence and multiple exposure images. The images used in the manuscript are in the red boxes.

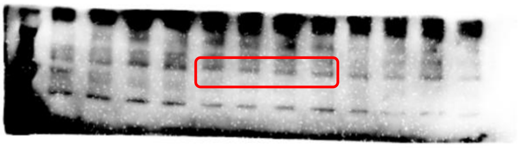

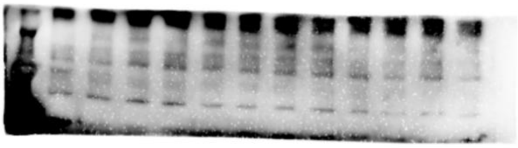

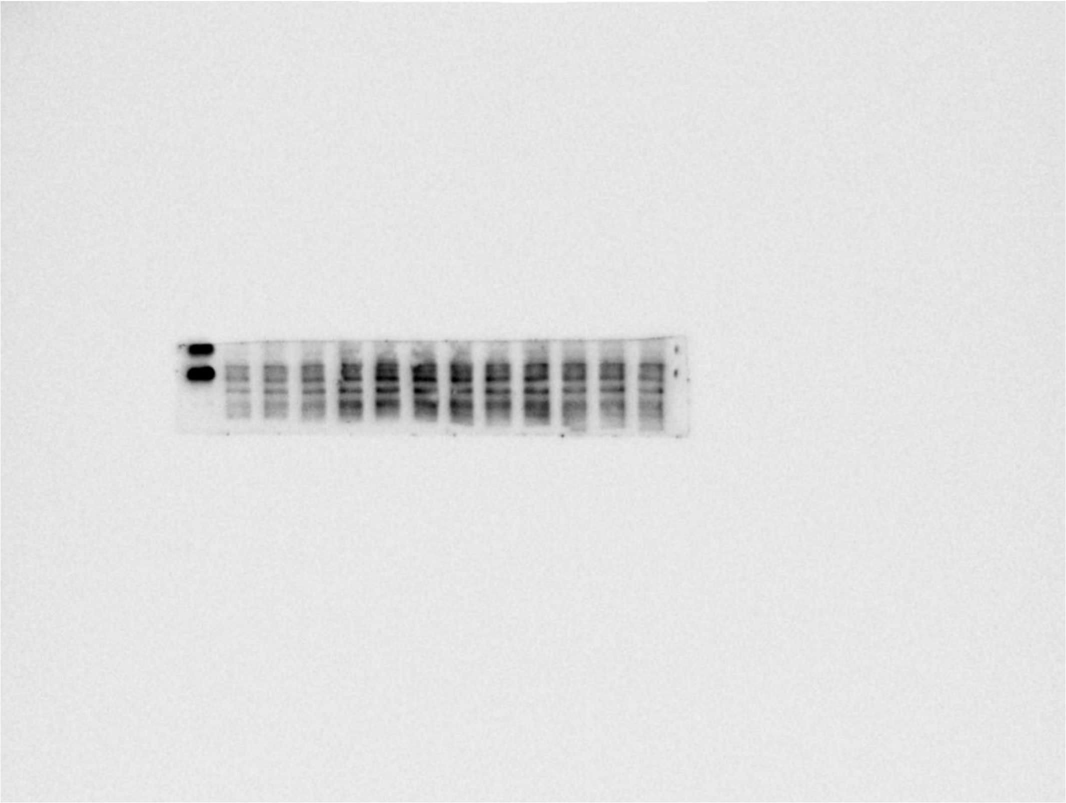

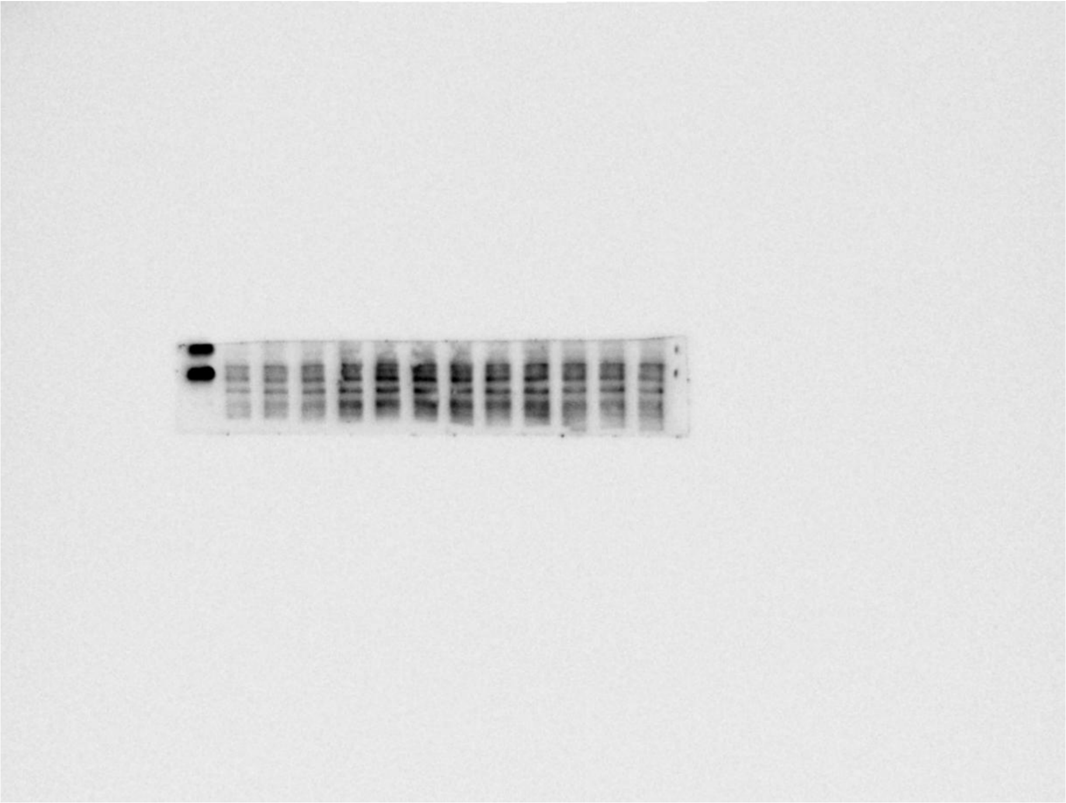

Figure 8A Original p-LSD1 gel blotting image. There were three repeated blots in sequence and multiple exposure images. The images used in the manuscript are in the red boxes.

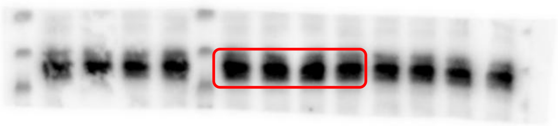

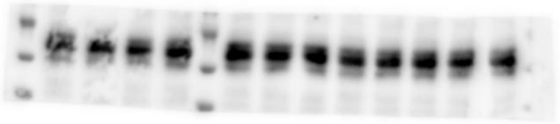

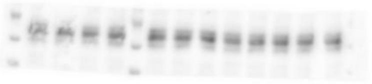

Figure 8B Original actin gel blotting image. There were three repeated blots in sequence and multiple exposure images. The images used in the manuscript are in the red boxes.

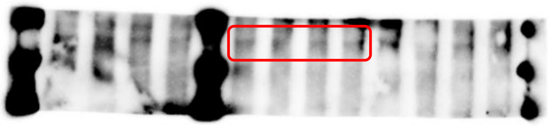

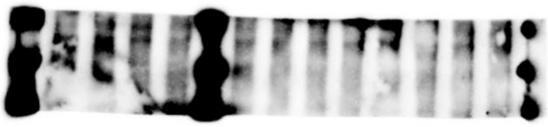

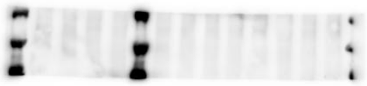

Figure 8B Original p-LSD1 gel blotting image. There were three repeated blots in sequence and multiple exposure images. The images used in the manuscript are in the red boxes.

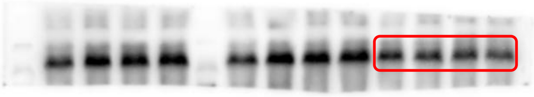

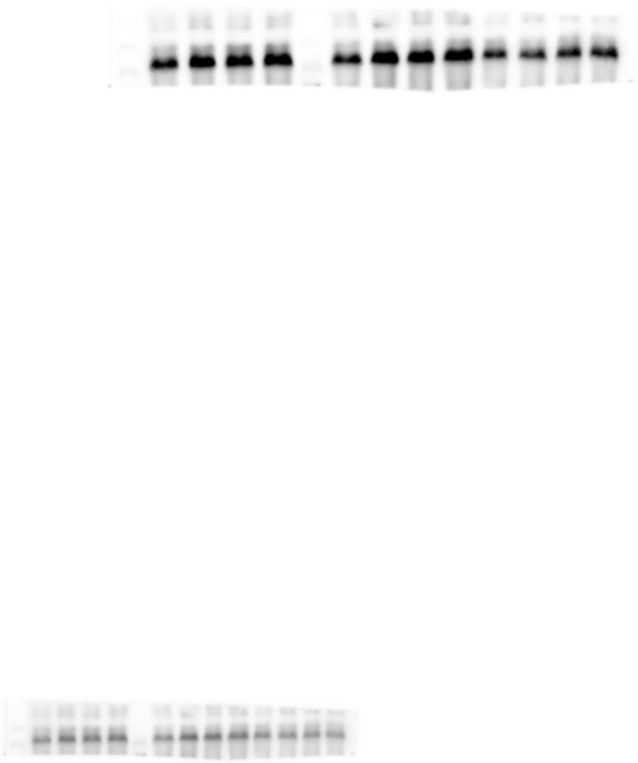

Figure 8C Original actin gel blotting image. There were three repeated blots in sequence and multiple exposure images. The images used in the manuscript are in the red boxes.

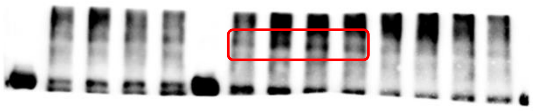

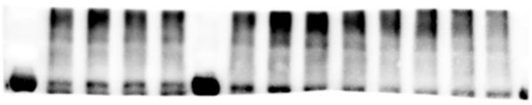

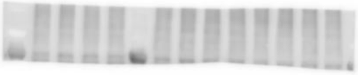

Figure 8C Original p-LSD1 gel blotting image. There were three repeated blots in sequence and multiple exposure images. The images used in the manuscript are in the red boxes.

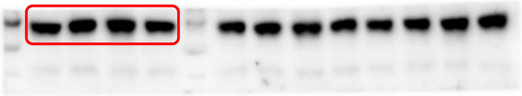

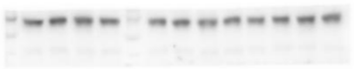

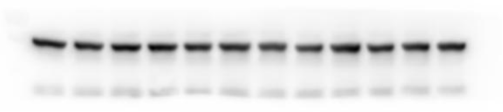

Figure 8D Original actin gel blotting image. There were three repeated blots in sequence and multiple exposure images. The images used in the manuscript are in the red boxes.

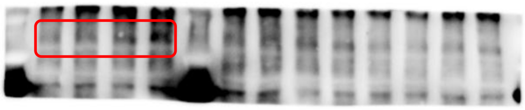

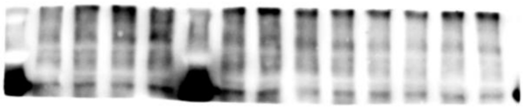

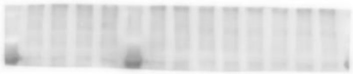

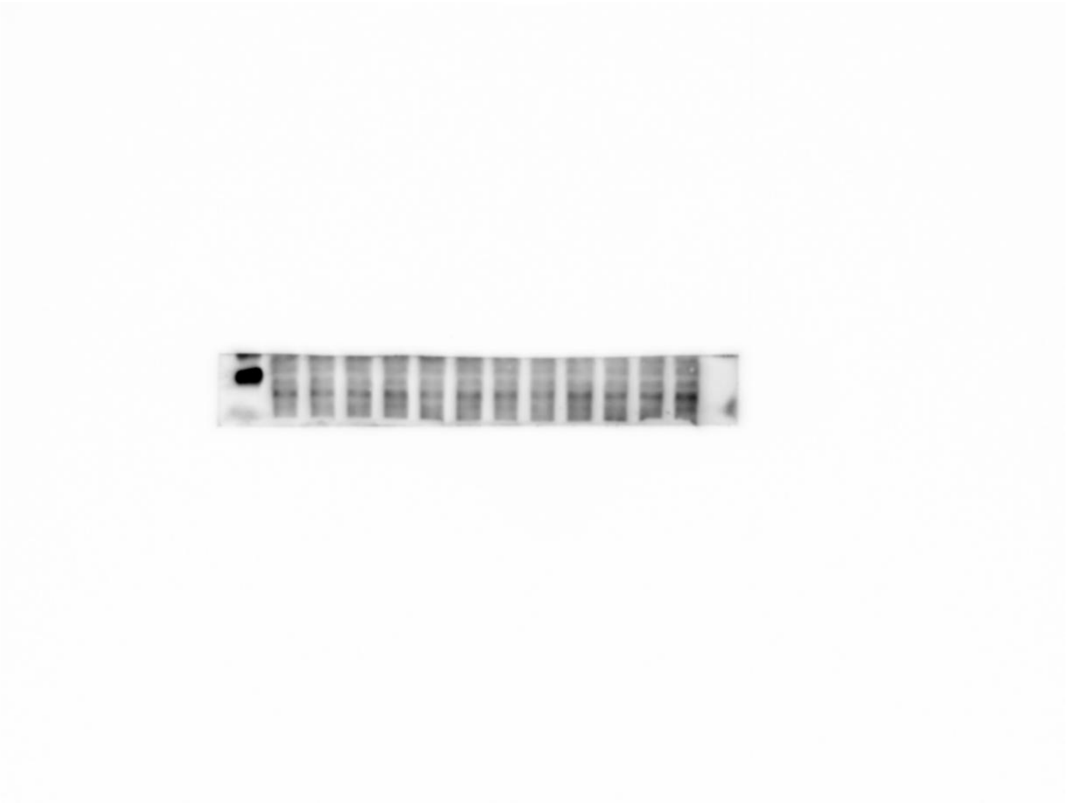

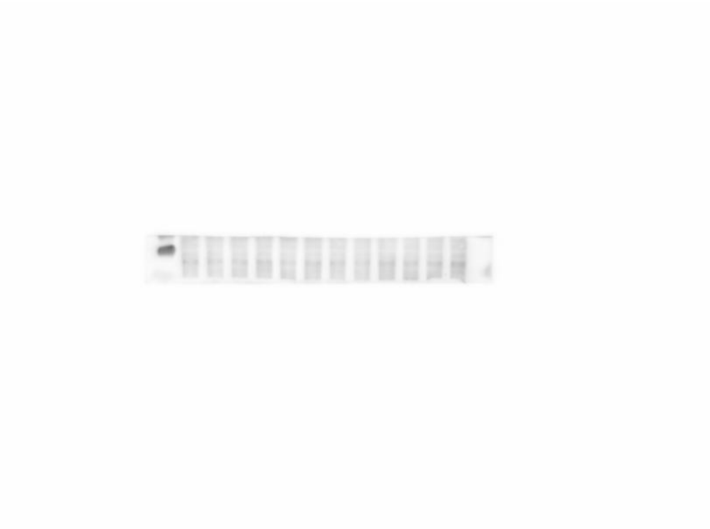

Figure 8D Original p-LSD1 gel blotting image. There were three repeated blots in sequence and multiple exposure images. The images used in the manuscript are in the red boxes.

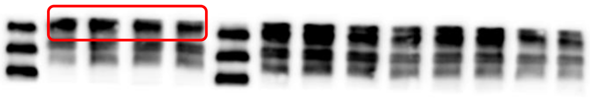

Figure 9A Original actin gel blotting image and all replication，the images used in the manuscript are in the red boxes.

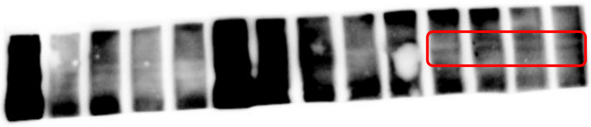

Figure 9A Original p-LSD1 gel blotting image and all replication，the images used in the manuscript are in the red boxes.

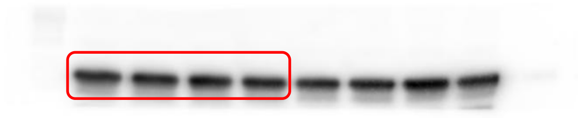

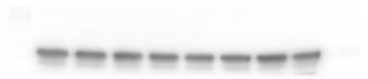

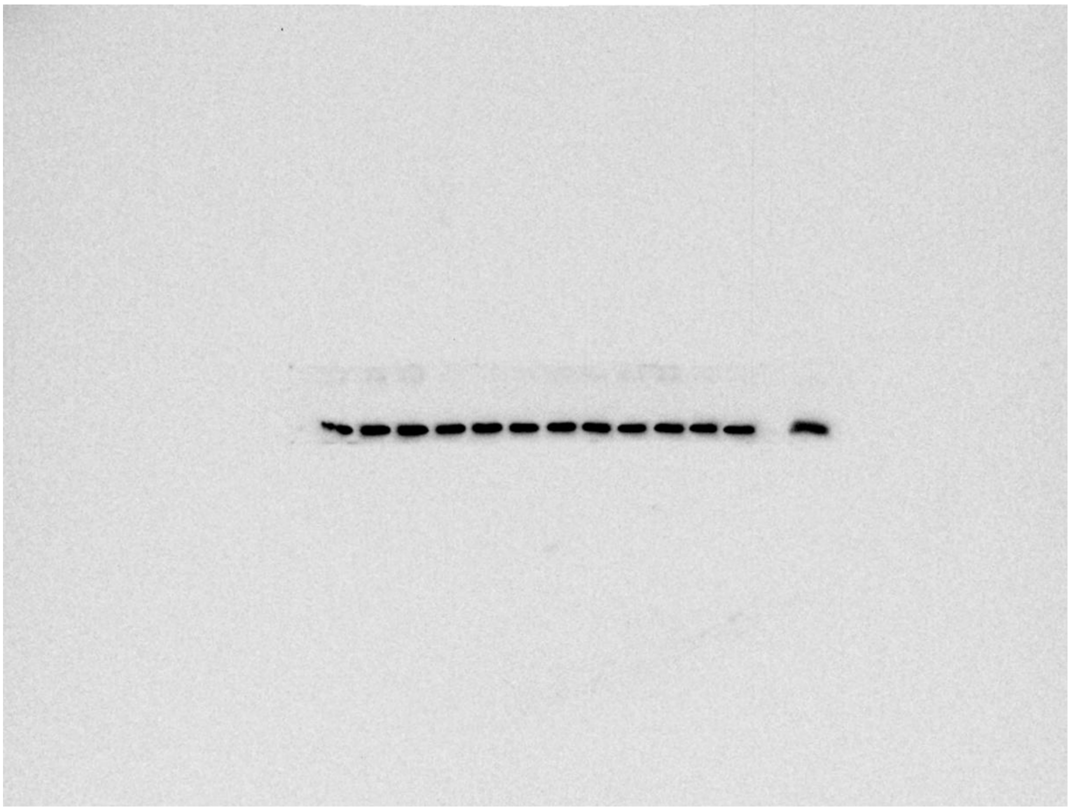

Figure 9B Original actin gel blotting image. There were three repeated blots in sequence and multiple exposure images. The images used in the manuscript are in the red boxes.

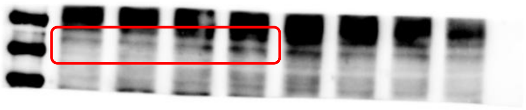

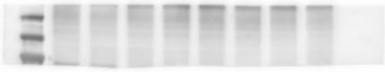

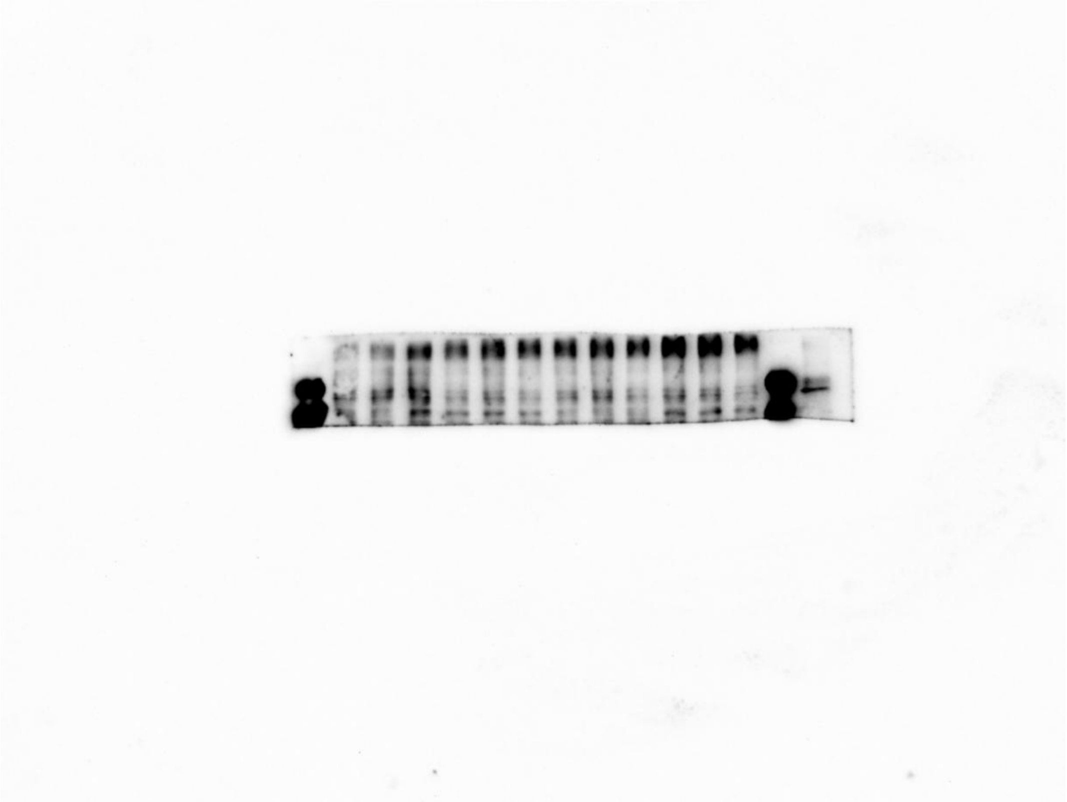

Figure 9B Original p-LSD1 gel blotting image. There were three repeated blots in sequence and multiple exposure images. The images used in the manuscript are in the red boxes.

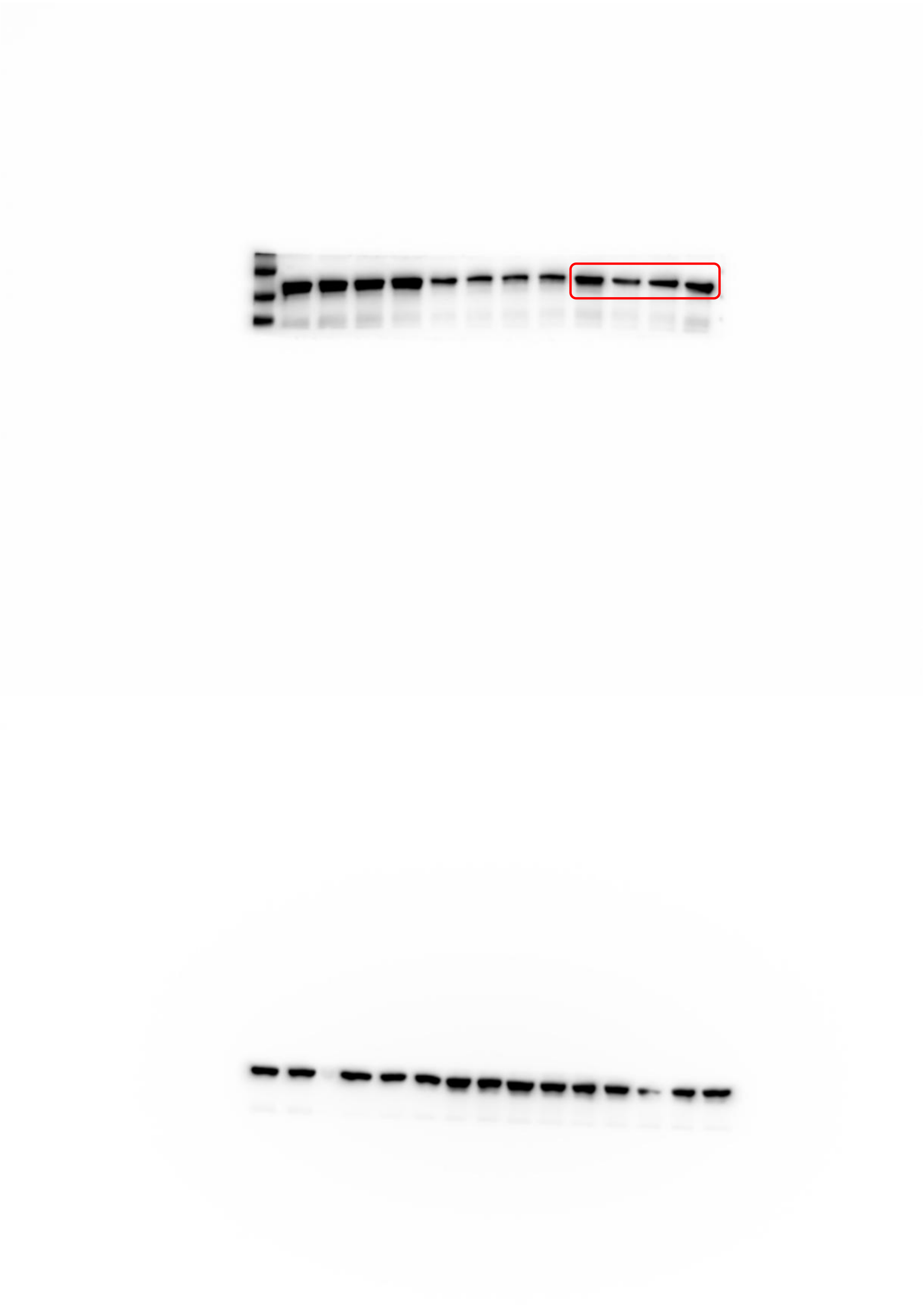

Figure 9C Original actin gel blotting image all replication，the images used in the manuscript are in the red boxes.

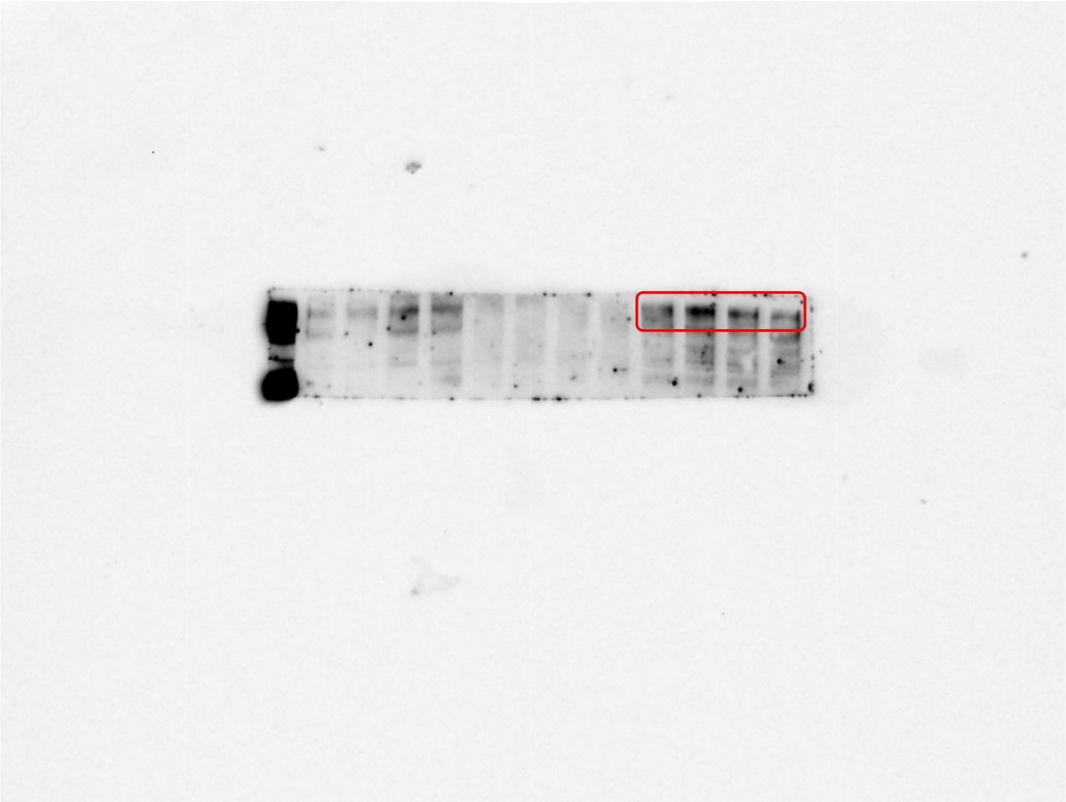

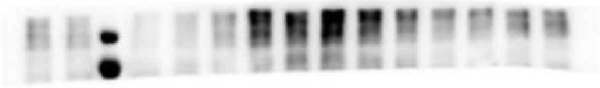

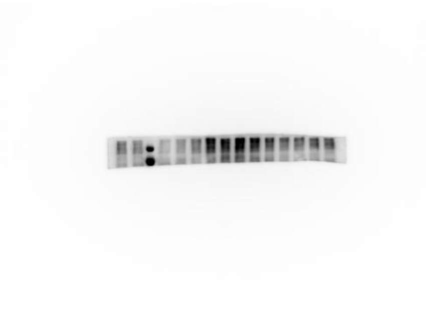

Figure 9C Original p-LSD1 gel blotting image. There were three repeated blots in sequence and multiple exposure images. The images used in the manuscript are in the red boxes.

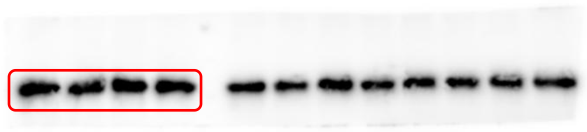

Figure 9D Original actin gel blotting image. There were three repeated blots in sequence and multiple exposure images. The images used in the manuscript are in the red boxes.

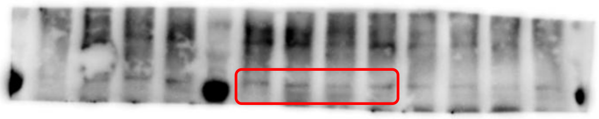

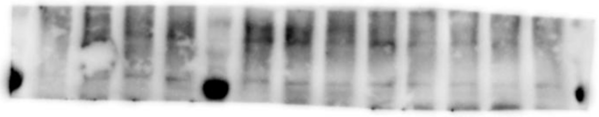

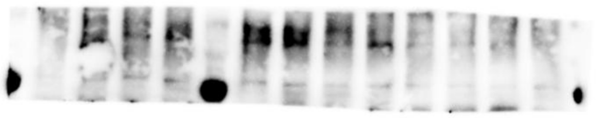

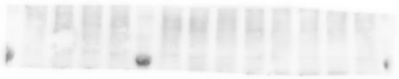

Figure 9D Original p-LSD1 gel blotting image. There were three repeated blots in sequence and multiple exposure images. The images used in the manuscript are in the red boxes.

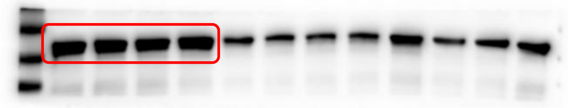

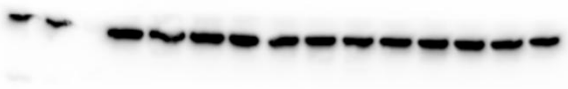

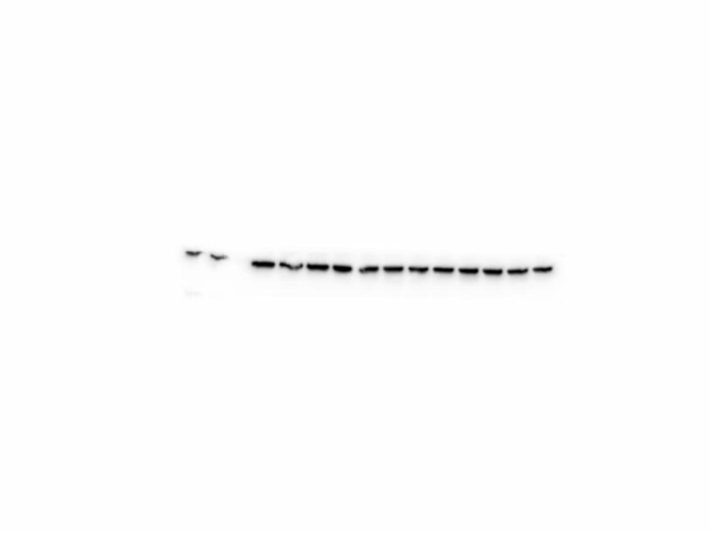

Figure 10A Original actin gel blotting image. There were three repeated blots in sequence and multiple exposure images. The images used in the manuscript are in the red boxes.

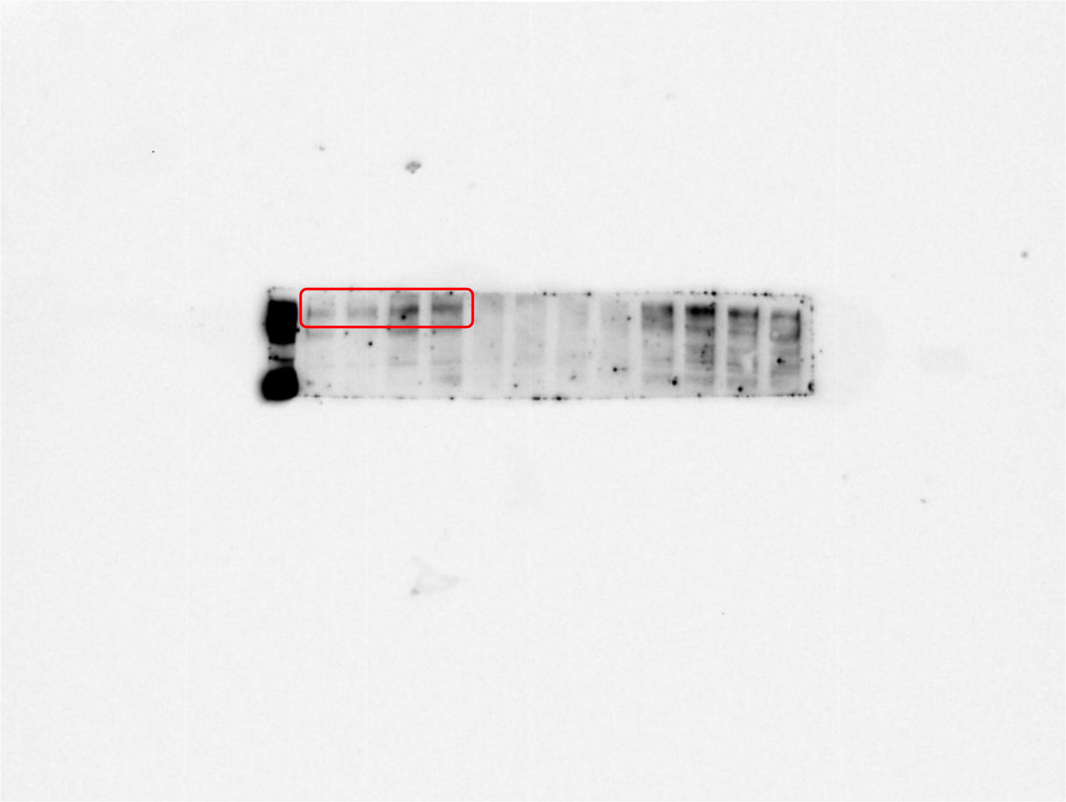

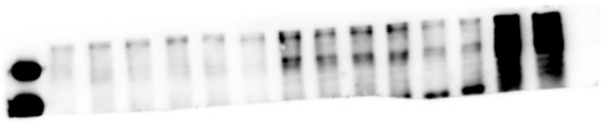

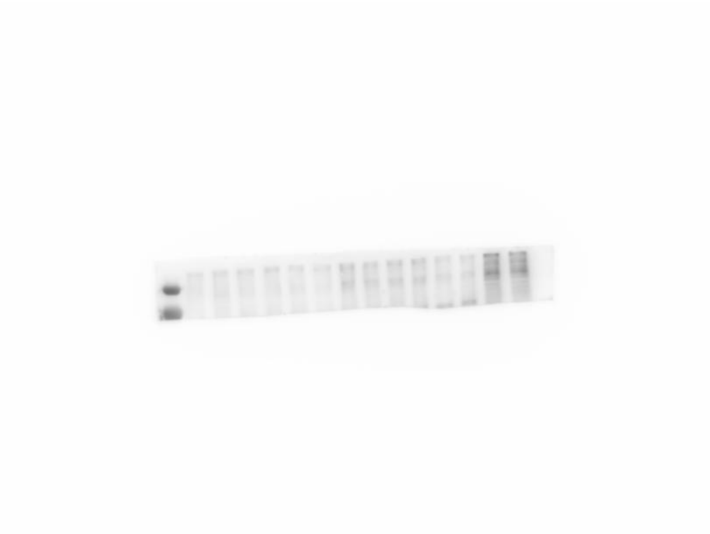

Figure 10A Original p-LSD1 gel blotting image. There were three repeated blots in sequence and multiple exposure images. The images used in the manuscript are in the red boxes.

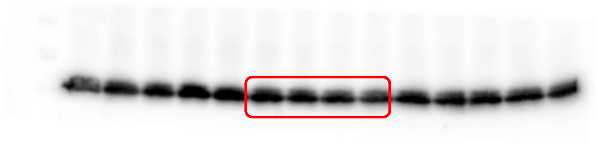

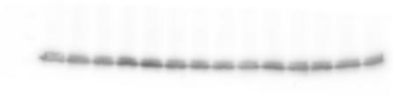

Figure 10B Original actin gel blotting image. There were three repeated blots in sequence and multiple exposure images. The images used in the manuscript are in the red boxes.

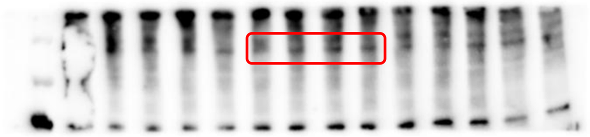

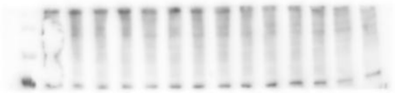

Figure 10B Original p-LSD1 gel blotting image. There were three repeated blots in sequence and multiple exposure images. The images used in the manuscript are in the red boxes.

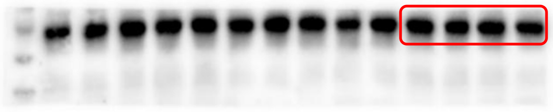

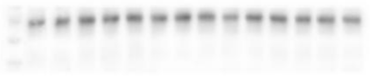

Figure 10C Original actin gel blotting image. There were three repeated blots in sequence and multiple exposure images. The images used in the manuscript are in the red boxes.

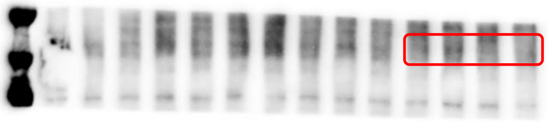

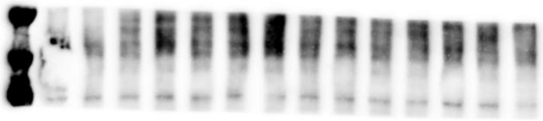

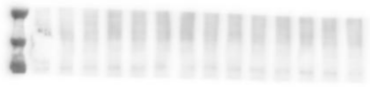

Figure 10C Original p-LSD1 gel blotting image. There were three repeated blots in sequence and multiple exposure images. The images used in the manuscript are in the red boxes.

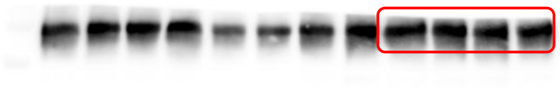

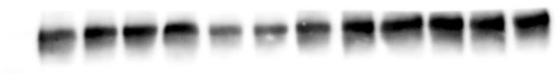

Figure 10D Original actin gel blotting image. There were three repeated blots in sequence and multiple exposure images. The images used in the manuscript are in the red boxes.

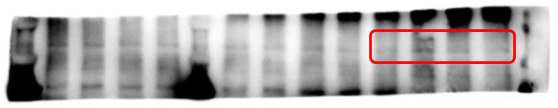

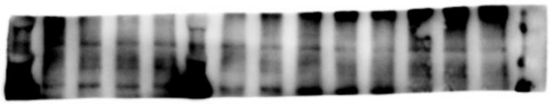

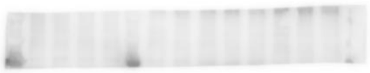

Figure 10D Original p-LSD1 gel blotting image There were three repeated blots in sequence and multiple exposure images. The images used in the manuscript are in the red boxes.
